# *Hepatincolaceae* (*Alphaproteobacteria*) are Distinct From *Holosporales* and Independently Evolved to Associate With Ecdysozoa

**DOI:** 10.1111/1462-2920.70028

**Published:** 2025-01-10

**Authors:** Michele Castelli, Leandro Gammuto, Diona Podushkina, Matteo Vecchi, Tiziana Altiero, Emanuela Clementi, Roberto Guidetti, Lorena Rebecchi, Davide Sassera

**Affiliations:** ^1^ Department of Biology and Biotechnology University of Pavia Pavia Italy; ^2^ Dipartimento di Scienze Della Vita Università degli Studi di Modena e Reggio Emilia Modena Italy; ^3^ Institute of Systematics and Evolution of Animals Polish Academy of Sciences Krakow Poland; ^4^ Dipartimento Educazione e Scienze Umane Università degli Studi di Modena e Reggio Emilia Modena Italy; ^5^ Fondazione IRCCS Policlinico San Matteo Pavia Italy

**Keywords:** bacterial parasites, gut bacteria, prophages, protists, *Rickettsiales*, symbiosis, tardigrades

## Abstract

The *Hepatincolaceae* (*Alphaproteobacteria*) are a group of bacteria that inhabit the gut of arthropods and other ecdysozoans, associating extracellularly with microvilli. Previous phylogenetic studies, primarily single‐gene analyses, suggested their relationship to the *Holosporales*, which includes intracellular bacteria in protist hosts. However, the genomics of *Hepatincolaceae* is still in its early stages. In this study, the number of available *Hepatincolaceae* genomes was increased to examine their evolutionary and functional characteristics. It was found that the previous phylogenetic grouping with *Holosporales* was incorrect due to sequence compositional biases and that *Hepatincolaceae* form an independent branch within the *Hepatincolaceae*. This led to a reinterpretation of their features, proposing a new evolutionary scenario that involves an independent adaptation to host association compared to the *Holosporales*, with distinct specificities. The *Hepatincolaceae* exhibit greater nutritional flexibility, utilising various molecules available in the host gut and thriving in anaerobic conditions. However, they have a less complex mechanism for modulating host interactions, which are likely less direct than those of intracellular bacteria. In addition, representatives of *Hepatincolaceae* show several lineage‐specific traits related to differences in host species and life conditions.

## Graphical abstract

The main differences between the ‘*Ca*. Hepatincolaceae’ and the *Holosporales* are their habitats and modes of existence. The former are non‐motile bacteria that exist extracellularly in the host gut lumen, associating with microvilli, while the latter are intracellular bacteria, primarily hosted by protists such as amoebae and ciliates, and often possess flagellar genes.

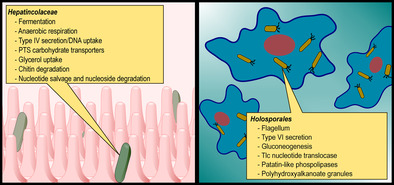

## 1 Introduction

Associations between prokaryotes and eukaryotes are widespread and diverse (McFall‐Ngai et al. 2013; Drew, Stevens, and King 2021; Husnik et al. 2021). Many phylogenetically and physiologically diverse bacteria have evolved metabolic dependence on their hosts, accompanied by marked genome size reduction (McCutcheon and Moran 2012; Bennett and Moran 2015; Wernegreen 2017).

In particular, the *Alphaproteobacteria* encompass multiple obligate host‐associated lineages (Hördt et al. 2020), including the *Rickettsiales* and the *Holosporales* (Muñoz‐Gómez et al. 2019; Castelli et al. 2024). Curiously, early molecular phylogenies on 16S rRNA gene sequences lumped those two lineages together, with the *Holosporales* as early diverging among the *Rickettsiales* (e.g., Wang et al. 2004). More recent analyses based on multiple markers allowed to establish that this grouping was artefactual (Georgiades et al. 2011; Ferla et al. 2013; Schulz et al. 2014; Wang and Wu 2015), due to convergently evolved sequence compositional biases (i.e., low GC content) as a consequence of obligate associations with their hosts (Muñoz‐Gómez et al. 2019). Specifically, the *Rickettsiales* are an ancient and early diverging lineage within *Alphaproteobacteria* (Wang and Luo 2021), while the *Holosporales* have originated independently and more recently within the *Rhodospirillales sensu* Hördt et al. (2020). Based on this phylogenetic position, it was even proposed to lower *Holosporales* at the family rank within *Rhodospirillales* (Muñoz‐Gómez et al. 2019). Here, we will follow the definition of the order *Holosporales* by Szokoli et al. (2016) since it allows for the focus on the diversity and evolution of its main sub‐lineages, which are accordingly ranked as families.

The *Holosporales* include four families (Hess, Suthaus, and Melkonian 2016; Szokoli et al. 2016; Schrallhammer, Castelli, and Petroni 2018). Three of them, namely *Holosporaceae*, ‘*Caedimonadaceae*’, and ‘*Candidatus* Paracaedibacteraceae’ (from now on, *Candidatus* will be abbreviated as *Ca*.), encompass bacteria that are intracellularly associated with their eukaryotic hosts, typically protists (Horn et al. 1999; Birtles et al. 2000; Baker et al. 2003; Dirren and Posch 2016; Hess, Suthaus, and Melkonian 2016; Szokoli et al. 2016; Potekhin et al. 2018; Boscaro et al. 2019; George et al. 2020; Midha et al. 2021; Zilio et al. 2021; Castelli et al. 2022; Shiohama et al. 2022; Lanzoni et al. 2024), with few exceptions of arthropods (Jones, McCormick, and Martin 2008; Nunan et al. 2013; Konecka and Olszanowski 2019). These protist‐associated *Holosporales* include very peculiar representatives such as infectious ones (Schulz et al. 2014; Fokin et al. 2019; Beliavskaia et al. 2020; Schrallhammer and Potekhin 2020), and others able to kill uninfected hosts (Schrallhammer and Schweikert 2009; Schrallhammer, Castelli, and Petroni 2018). In the last few years, the representatives of these families have been the subject of multiple genomic and phylogenomic studies (Georgiades et al. 2011; Wang and Wu 2015; George et al. 2020; Midha et al. 2021; Castelli et al. 2022; Shiohama et al. 2022; Giovannini, Petroni, and Castelli 2024).

On the other hand, members of the fourth family (named ‘*Ca*. Hepatincolaceae’ (Szokoli et al. 2016) or ‘*Ca*. Tenuibacteraceae’ (Kroer et al. 2016); from now on, *Hepatincolaceae*) have been found so far hosted only by ecdysozoan animals. Such hosts are mostly arthropods, including insects (Ramírez‐Puebla et al. 2010; Koch et al. 2013), crustaceans (Wang et al. 2004; Li et al. 2007; Bauermeister, Ramette, and Dattagupta 2012; Dittmer et al. 2023), arachnids (Qu et al. 2015) and myriapods (Chipman et al. 2014), as well as tardigrades (Vecchi et al. 2018; Guidetti et al. 2020) and priapulids (Kroer et al. 2016). Based on the available data, the *Hepatincolaceae* dwell within the host gut lumen (Li et al. 2007; Koch et al. 2013; Bouchon, Zimmer, and Dittmer 2016), being extracellularly associated with host microvilli (Wang et al. 2004; Kroer et al. 2016) and likely exploiting this condition to scavenge nutrients (Dittmer et al. 2023).

To sum up, *Hepatincolaceae* are quite sharply distinguished from the other *Holosporales*, in particular, for host lineages and extracellular location. Phylogenetic analyses on 16S rRNA gene sequences most frequently placed them as early diverging within the *Holosporales* (Kroer et al. 2016; Guidetti et al. 2020; Lanzoni et al. 2024). However, they are still poorly investigated at the genomic and phylogenomic level, as the first three genome sequences of this family, all belonging to the genus ‘*Ca*. Hepatincola’ (from now on, *Hepatincola*), were only recently published (Dittmer et al. 2023).

Here, we present an extended genome sampling of the *Hepatincolaceae*, which allowed us to infer a novel phylogenetic and evolutionary framework. Specifically, we newly sequenced the symbiont of the tardigrade *Richtersius* cf. *coronifer* (for a better definition of the tardigrade taxon and its symbionts see Guidetti et al. 2016, 2020; Vecchi et al. 2018), and assembled the genome of the symbiont of the myriapod *Strigamia maritima* from reads that were previously obtained for sequencing the genome of its host (Chipman et al. 2014). Moreover, we determined that a previously published genomic assembly of a bacterial symbiont of the termite *Labiotermes labralis* (Hervé et al. 2020) can be ascribed to the *Hepatincolaceae*, so we included it in our analyses. Leveraging this extended sampling and accounting for compositional heterogeneity biases, we re‐assessed the phylogenetic positioning of the *Hepatincolaceae*, determining that they are phylogenetically distinct from the ‘true’ *Holosporales sensu* Szokoli et al. (2016), thus representing an evolutionarily independent obligatorily host‐associated lineage within the *Rhodospirillales sensu* Hördt et al. (2020).

## 2 Materials and Methods

### 2.1 Genome Sequencing, Assembly and Annotation of the Symbiont of *R.* cf. *coronifer*

The starting point was a *R*. cf. *coronifer* population originally isolated from the moss in Öland, Sweden (Vecchi et al. 2018). DNA extraction was conducted from multiple single individuals, as previously described (Guidetti et al. 2020). Then, semi‐quantitative PCR (i.e., on original samples and the respective serial 1:10 dilutions) was performed with previously defined primers and cycling conditions for the SSU rRNA genes of the bacterium (Guidetti et al. 2020) and of its host (Bertolani et al. 2014), respectively. Accordingly, the specimen with the highest estimated bacterium/host ratio was subjected to whole‐genome amplification with the REPLI‐g Single Cell Kit (QIAGEN). Then, total DNA sequencing was carried out through a Nextera XT library on an Illumina HiSeq X machine by Admera Health (South Plainfield, NJ, USA), producing 44,276,484 pairs of 150 bp reads.

After an assessment with FastQC (Andrews 2010) that confirmed the high quality of the reads, these were directly assembled with SPAdes 3.6 (Bankevich et al. 2012), thus obtaining 86,693 contigs (296,744,432 bp). The choice of SPAdes over other software more tailored for metagenomes (e.g., metaSPAdes [Nurk et al. 2017]) was due to a previous successful experience in alike host–symbiont datasets (e.g., Castelli et al. 2019, 2022, 2024; Giovannini, Petroni, and Castelli 2024). After this preliminary assembly, a multi‐step procedure was applied (Castelli et al. 2019), to select the sequences belonging to the bacterial symbiont based on the blobology pipeline (Kumar et al. 2013). This approach, feasible in low‐scale studies such as herein, allows an effective and accurate genome assembly of bacteria from low‐complexity metagenomes, such as the analysed herein host metagenomes (see also Section 3 for estimates on genome completeness and contamination). Accordingly, the contigs were classified as per their length, GC% content, NCBI taxonomy of the best megablast hit on the NCBI nucleotide database, and sequencing coverage based on reads mapped on the novel assembly with Bowtie2 (Langmead and Salzberg 2012). Considering also the rRNA genes identified with barrnap (Table S1) (Seemann 2013), a selection of contigs with coverage higher than 100 was performed (Figure S1), and the respective mapping reads (Langmead and Salzberg 2012) were reassembled separately with SPAdes, using the option ‐k 21,33,55,77,99,121. The selected contig sequences were manually examined and revised prior to and after reassembly by examining blastp hits on NCBI nr protein database after annotation with Prokka 1.10 (Seemann 2014), and using Bandage (Wick et al. 2015) for visualisation of SPAdes assembly graphs.

Moreover, to rule out the presence of additional sequences belonging to the symbiont, the annotated ORFs on contigs with coverage 10–100 were queried on the NCBI nr protein database with DIAMOND (Buchfink, Xie, and Huson 2015), applying an e‐value threshold of 1e−5. DIAMOND results for contigs with at least one ORF with the best hit on *Bacteria* were inspected manually.

Then, primers were designed on selected contig ends (Table S2), and PCR reactions were performed with the Takara ExTaq (Takara Bio, Japan) or with the PCRBIO VeriFi polymerase (PCR Biosystems, London, UK). Products were Sanger sequenced at Eurofins Genomics (Ebersberg, Germany).

The final genome assembly was annotated with Prokka, and results were manually curated by inspecting the blastp hits of the predicted proteins on NCBI nr and on a custom database of *Holosporales*, as well as on NCBI conserved domains (Lu et al. 2020).

### 2.2 Selection and Assembly of the *Hepatincolaceae* Symbiont of *S. maritima*

We aimed to expand the set of genome assemblies of *Hepatincolaceae* from published sequencing data besides the three available *Hepatincola* genomes (Dittmer et al. 2023). The first strategy involved inspection of the SILVA taxonomy (release 138.1) (Quast et al. 2013) and the reference 16S rRNA gene tree in ARB (Ludwig et al. 2004), looking for relatives of *Hepatincola porcellionum* (AY188585). Accordingly, a 16S rRNA gene sequence was identified (AFFK01003480) as being derived from the genome assembly project of the myriapod *S. maritima* (Chipman et al. 2014), suggesting the presence of a putative *Hepatincolaceae* symbiont. The corresponding reads (SRX326837, SRX326839, SRX326840 and SRX326841) were downloaded, combined and processed to selectively assemble the bacterial genome as described above for the symbiont of *R*. cf. *coronifer*, with some differences, presented below. The preliminary assembly counted 600,069 contigs (248,668,256 bp). In this case, to increase the specificity and sensitivity of the taxonomic assignment, a DIAMOND blastx search of the whole set of preliminary contigs on NCBI nr was used instead of a blastn search on NCBI nucleotide. The contigs with log_10_ coverage lower than 1.875, excluding those with best hits on eukaryotic sequences, were selected (Figure S2), and the corresponding mapped reads were reassembled with default SPAdes settings. The resulting reassembly was manually curated as described above. The second strategy consisted in testing the affiliated of published assemblies, and is described in the section 2.3.

### 2.3 Phylogenomic Analyses

A comprehensive phylogenomic analysis was conducted to investigate the phylogeny of the *Hepatincolaceae*, including assigning potential published Metagenome‐Assembled Genomes (MAGs) to this lineage and clarifying the position within *Alphaproteobacteria* of the *Hepatincolaceae* as a whole. For this purpose, the two assemblies obtained in this study and the three *Hepatincola* genomes (Dittmer et al. 2023) were merged into a representative set of *Alphaproteobacteria*. This set was obtained by taking genome assemblies from described alphaproteobacterial lineages (plus other *Proteobacteria* as outgroup) from a previous study (Castelli et al. 2024) (with the exclusion of *Rickettsiales* and ‘*Ca*. Pelagibacterales’, known to be involved in phylogenetfic artefacts with *Holosporales* due to compositional biases and fast sequence evolution rates; Muñoz‐Gómez et al. 2019) and a selection of additional assemblies (in particular MAGs) belonging to yet undescribed alphaproteobacterial lineages based on the GTDB taxonomy (r207 release) (Parks et al. 2022) (Table S3). The final set counted 179 organisms. This approach also allowed us to recognise that an additional published assembly (Hervé et al. 2020) is ascribable to the *Hepatincolaceae* (see Section 3 for details). The completeness and contamination degree of all *Hepatincolaceae* and alphaproteobacterial MAGs were verified with CheckM 1.2.3 (Parks et al. 2015).

Annotated protein sequences were predicted for each assembly in the dataset with Prokka. Phylogenomics was performed with a previously determined dataset of 179 alphaproteobacterial orthologs (Castelli et al. 2024), selected from eggNOG orthogroups (Huerta‐Cepas et al. 2019) predicted with eggNOG mapper (Cantalapiedra et al. 2021) as previously described (Castelli et al. 2024) (Table S4). The sequences of each of the identified 179 orthologs of interest were aligned with MAFFT 7.475 L‐INS‐i (Katoh and Standley 2013), and trimmed with BMGE 1.12 (Criscuolo and Gribaldo 2010) selecting the BLOSUM30 matrix, as recommended in the BMGE manual for distantly related sequences. Trimmed orthologs were concatenated together (Borowiec 2016). To account for potential artefacts due to compositional heterogeneity, the 10%, 20%, 30%, 40% or 50% most biassed sites, identified as previously described (Muñoz‐Gómez et al. 2019), were removed from the concatenated alignments. Maximum likelihood phylogenies were inferred on the original concatenated alignment and each compositionally trimmed alignment with IQ‐TREE 1.6.12 (Nguyen et al. 2015) with the LG + C60 + F + R6 model as in (Muñoz‐Gómez et al. 2019), performing 1000 ultra‐fast bootstraps (Minh, Nguyen, and von Haeseler 2013) and SH‐aLRT with 1000 replicates.

Average nucleotide identity (ANI) values among the *Hepatincolaceae* were calculated with the EZBioCloud tool (Yoon et al. 2017). Moreover, the assignment of the *Hepatincolaceae* to the GTDB taxonomic groups was obtained with GTDB‐Tk 2.1.1 (Chaumeil et al. 2020).

### 2.4 Investigations on *Hepatincolaceae* Bacteria in the Microbiome of Tardigrades

To screen for the presence of relatives of the symbiont of *R*. cf. *coronifer* in previously published tardigrade microbiomes, we respectively downloaded already clustered OTUs, when available (Tibbs‐Cortes, Tibbs‐Cortes, and Schmitz‐Esser 2022; Zawierucha et al. 2022), or sequencing reads (Kaczmarek et al. 2020; Mioduchowska et al. 2021; Boscaro et al. 2022) (Table S5). Reads were processed with QIIME2 (Bolyen et al. 2019) and de novo clustered into OTUs with 99% identity. OTU sequences aligned > 90% of their length with at least 97% identity to the 16S rRNA gene of the symbiont of *R*. cf. *coronifer* were further manually verified by comparison with the NCBI nr database to assess their affiliation. For the phylogenetic analyses, 56 sequences of *Hepatincolaceae* plus 10 *Rhodospirillales* as outgroups were automatically aligned with ARB 5.5 on the SSU ref. NR99 SILVA database 138.1, manually edited to optimise base pairing in the predicted rRNA structure, and trimmed at the shortest sequence length at both sides (final 1128 sites). The optimal substitution model was identified using jModelTest 2.1 (Darriba et al. 2012) according to the Akaike information criterion. A maximum likelihood tree was inferred with PHYML 2.4 (Guindon and Gascuel 2003) performing 1000 pseudo‐replicates. Due to its short size, the retrieved OTU sequence related to the symbiont of *R*. cf. *coronifer* (see Section 3 for details) was added later using the quick‐add marked function of the ARB package.

### 2.5 Functional and Metabolic Predictions and Comparisons

Clusters of orthologous groups (COGs) was identified with the NCBI pipeline (Galperin et al. 2015) in the *Hepatincolaceae* (the newly assembled symbiont of *R*. cf. *coronifer* and symbiont of *Strigamia*, the three *Hepatincola* spp., and the symbiont of *L. labralis* GCA_009780035.1), their closest relatives based on phylogeny ( *Thalassospira profundimaris* , *Terasakiella pusilla* , symbiont of *Haliotis discus hannai* , GCA_001510075.1, GCA_001830425.1, GCA_002327565.1, GCA_002687515.1, GCA_009694195.1, GCA_009649675.1, GCA_013204045.1, GCA_014859895.1, GCA_018662225.1) and a representative set of *Holosporales* ( *Holospora undulata* , ‘*Ca*. Hepatobacter penaei’, ‘*Ca*. Bealeia paramacronuclearis’, ‘*Caedimonas varicaedens*’, ‘*Ca*. Nucleicultrix amoebiphila’, ‘*Ca*. Paracaedibacter acanthamoebae’, ‘*Ca*. Odyssella thessalonicensis’, ‘*Ca*. Finniella inopinata’). The COG repertoire for each organism (or group of organisms) is herein defined as the number of unique COGs annotated in the respective gene set(s), regardless of how many genes were annotated to each COG or if some gene was annotated to multiple COGs. Such repertoires were then compared manually, in particular among *Hepatincolaceae* and between *Hepatincolaceae* and the other organisms (see Section 3 and Text S1 for details). Reference metabolic pathways for such comparisons were taken from BioCyc (Karp et al. 2019) and KEGG (Kanehisa et al. 2016).

## 3 Results

### 3.1 Novel Genomes of *Hepatincolaceae* Bacteria

In this work, we newly sequenced and assembled the genome of the *Hepatincolaceae* symbiont of the tardigrade *R*. cf. *coronifer* (Guidetti et al. 2020), and assembled the genome of the *Hepatincolaceae* symbiont of the centipede *S. maritima* from previously published reads (Chipman et al. 2014). Both assemblies had sizes and GC content comparable to the previously published genomes of *Hepatincola* (~1.3 Mb, and ~30 GC%; Table [Link], [Link]). The genome assembly of the symbiont of *Richtersius* was quite contiguous (15 contigs, N50 = 996,816 bp, L50 = 1), with no unconnected contig ends in the assembly graph, and few long repeats (thousands of bp), concentrated in two genome areas (Figure S3), which likely represent as many putative prophages (see below). This suggests that this assembly is likely complete and clean, consistent with the high completeness (98.9%) and no contamination (0.0%) estimated by CheckM (Table S7). On the other hand, the assembly of the symbiont of *Strigamia* is more fragmented (227 contigs, N50 = 7795 bp, L50 = 49), with a slightly lower completeness score (86.8%) and a slightly higher, though negligible, contamination (1.1%) (Table S7). This potential incompleteness can be explained by the relatively lower sequencing coverage in sequencing reads, which were obtained in a study focused on the genomics of the arthropod host (Chipman et al. 2014). This could also explain why some genes common in the other *Hepatincolaceae* could not be found in this bacterium (see Text S1).

### 3.2 Phylogenomics

We then aimed to infer the phylogenetic relationship among the five *Hepatincolaceae* with genome sequences available (i.e., the two novel ones and the three *Hepatincola* spp.; Dittmer et al. 2023), as well as of the *Hepatincolaceae* with respect to the *Holosporales* and other *Alphaproteobacteria*.

In the phylogeny obtained from the original concatenated alignment, all the *Hepatincolaceae* clustered together with full support (Figure S4). Moreover, a previously published MAG (GCA_009780035.1) was found to be nested within the same clade and was thus identified as a sixth additional member of the *Hepatincolaceae*. The respective sample originated from the gut of the soil‐feeding termite *L. labralis* (Hervé et al. 2020), which is comparable with the typical provenance of the members this bacterial lineage (i.e., gut of arthropods and other Ecdysozoa) (Wang et al. 2004; Kroer et al. 2016; Szokoli et al. 2016; Dittmer et al. 2023). This MAG belongs to the GTDB order and family WRAU01. Interestingly, GTDB‐Tk assigned to the same lineage also the other five *Hepatincolaceae* assemblies herein analysed, suggesting that WRAU01 could correspond to the *Hepatincolaceae*, and may be renamed accordingly in the future. The inner relationships of the *Hepatincolaceae* were fully supported as well, with *Hepatincola* Av and Pdp as closest relatives with respect to *Hepatincola* Pp, consistent with (Dittmer et al. 2023), the symbiont of *Labiotermes* as the sister group of *Hepatincola* spp., and the symbionts of *Richtersius* and *Strigamia* in progressive sequential branching order. The high sequence divergence of each of the latter three bacteria (ANI always below 68%) suggests that they should be ascribed to as many new separate genera and species (Table S8) (Barco et al. 2020). For the symbiont of *Richtersius*, this is consistent with previous analyses on the 16S rRNA gene (Guidetti et al. 2020). Screening of tardigrade microbiomes for relatives of the symbiont of *Richtersius* allowed the identification of a single OTU phylogenetically related to this bacterium (OTU000635: length: 257 bp, 16S rRNA gene identity: 98.81%), indicating the probable presence of a clade of tardigrade‐associated *Hepatincolaceae* (Figure S5). This OTU was retrieved in several samples from the same study (Tibbs‐Cortes, Tibbs‐Cortes, and Schmitz‐Esser 2022) involving multiple tardigrade species (Table S9). Accordingly, the symbiont of *Richtersius* will be from now on referred to as *Tardigradibacter bertolanii* (see taxonomic description at the end of Section 4).

**FIGURE 1:**
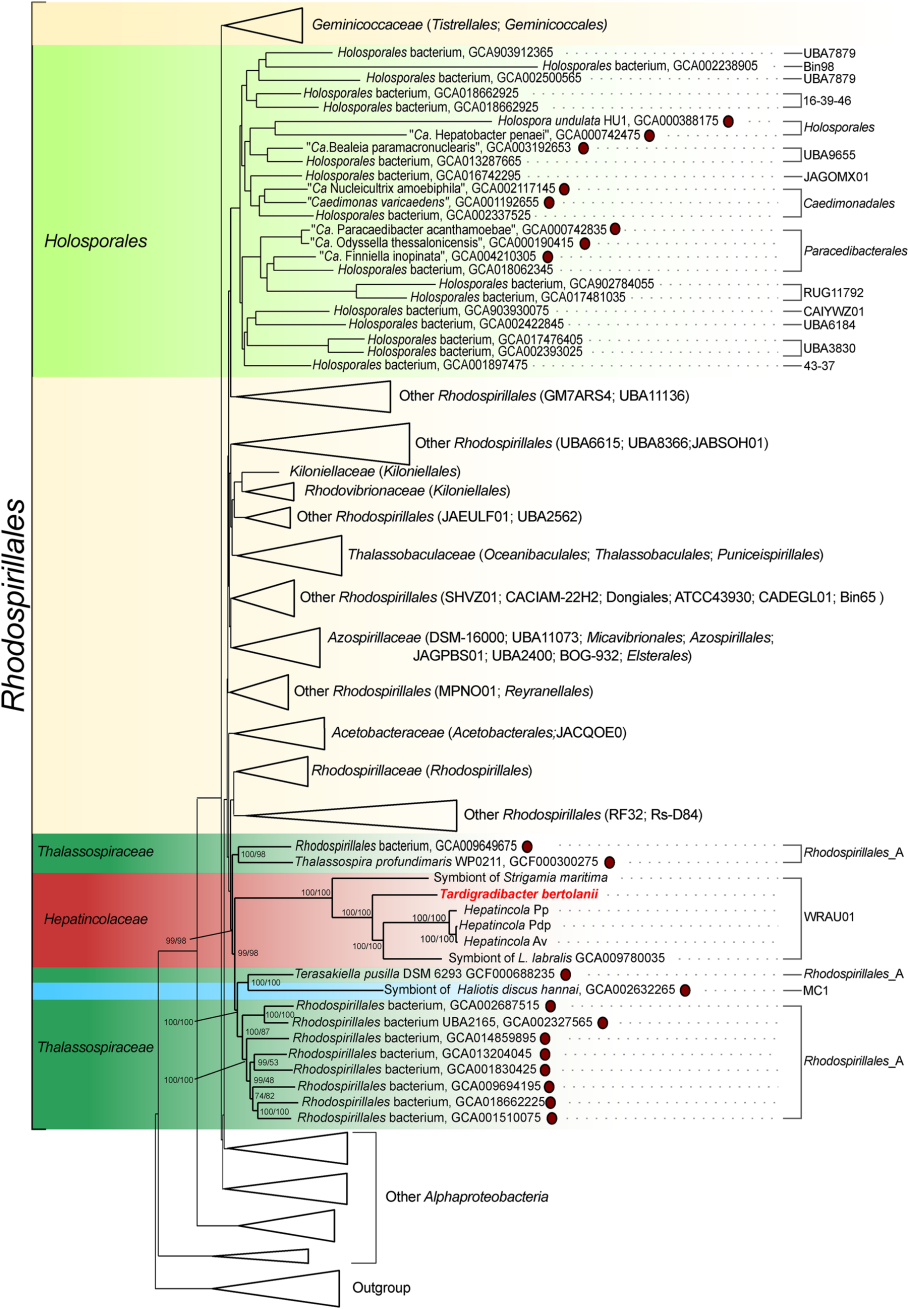
Phylogenetic tree of the *Alphaproteobacteria* after removing the 30% most compositionally biassed sites. Different coloured backgrounds highlight each group, namely the *Hepatincolaceae* (red), their free‐living relatives (dark green), the symbiont of *Haliotis* (blue), the *Holosporales* (light green), and the other *Rhodospirillales* (yellow). For space constraints, the families of the *Rhodospirillales* and the other more distantly related lineages are collapsed as triangular shapes (the full tree is shown in Figure S4, together with the tree inferred on the compositionally untreated dataset and those inferred with the other trimming thresholds). The assignments to order level groups from the GTDB taxonomy for each organism individually in the non‐collapsed lineages are reported on the right‐hand side, while those of the respective representatives are shown under parentheses for the collapsed lineages. The novel *Tardigradibacter bertolanii* is highlighted in bold, and all the non‐*Hepatincolaceae* genome assemblies employed in the comparative genomics are flanked by a dark red circle. For the clade encompassing the *Hepatincolaceae* and their relatives, the number of branches that stand for ultra‐fast bootstrap and SH‐aLRT support values are reported.

In the phylogenomic tree based on the original dataset, the *Hepatincolaceae* clade was retrieved in a quite early‐divergent position within *Alphaproteobacteria*, close to a number of assemblies from various origins, but distant from the *Holosporales* (Figure S4). We then aimed to counterbalance the effects of GC/AT compositional biases on the phylogenetic inference by progressively removing the most biassed sites from the alignment through an on‐purpose site selection method (Muñoz‐Gómez et al. 2019). When applying this approach, the *Hepatincolaceae* and their inner phylogenetic branches were stably supported, but the relationships with other *Alphaproteobacteria* drastically changed (Figures 1 and S4). Specifically, each of the alignments with 10% and 20% most biassed sites removed produced ‘unique’ topologies, probably indicative of partial and ‘ongoing’ attenuation of compositional biases (with the 20% trimming resulting in a close *Hepatincolaceae*–*Holosporales* relationship, comparable to previous reconstructions for those lineages; Kroer et al. 2016; Szokoli et al. 2016; Dittmer et al. 2023). Whereas, in the three trees obtained with the more pronounced trimming (30%, 40% and 50% most biassed sites removed), the *Hepatincolaceae* stably found the same novel phylogenetic ‘neighbourhood’ with high supports (always above 90% ultra‐fast bootstrap). Accordingly, the *Hepatincolaceae* branched within the *Rhodospirillales sensu* Hördt et al. (2020), similar to the *Holosporales*, but in a distinct and distantly related position. While the *Holosporales* (which include multiple groups labelled as orders in GTDB; Figures 1 and S4) were found closely related to the *Kiloniellaceae*, *Rhodovibrionaceae*, *Thalassobaculaceae* and *Azospirillaceae*, consistent with (Muñoz‐Gómez et al. 2019), the *Hepatincolaceae* were closely related to several genome assemblies originating from marine environments (Figures 1 and S4). In detail, the sister group of the *Hepatincolaceae* can be subdivided into two highly supported branches. The first branch is constituted of *T. pusilla* and a bacterial symbiont from the digestive gland of the mollusc *H. discus hannai* (Huang et al. 2020), while the second branch is constituted of eight marine MAGs. On the other hand, *T. profundimaris* and another marine MAG form the sister group of the lineage composed of the *Hepatincolaceae* and the closely related bacteria mentioned above. These relatives of the *Hepatincolaceae* are ascribed to the family *Thalassospiraceae* (and to the *Rhodospirillales*_A or MC‐1 order groups of GTDB; Figure 1). To sum up, the obtained topology showed a fully supported monophylum encompassing almost all the *Rhodospirillales sensu* Hördt et al. (2020) with monophyletic families (besides the inclusion of *Holosporales* and *Hepatincolaceae*), with the only independent branch represented by the *Geminicoccaceae*. These results, in particular the monophyly of this lineage with the exclusion of the latter family, are consistent with previous in‐depth analyses (Muñoz‐Gómez et al. 2019).

### 3.3 General Genome Comparisons

We compared the functional repertoire of the *Hepatincolaceae* with a selected set of other bacteria. These include their closest relatives among *Rhodospirillales*, as based on the novel phylogenomics with most biassed sites removed (Figure 1), namely genome assemblies and MAGs of marine bacteria, including the symbiont of *Haliotis*, being a closely related but independently evolved host‐associated bacterium. The set for comparison was completed by a representative selection of *Holosporales*, which had been considered the closest relatives of *Hepatincolaceae* until now. According to our analyses, the *Holosporales* and the *Hepatincolaceae* should be regarded as instances of independent evolution of host association involving evolutionarily related bacteria, thus making the comparison of their features useful to evidence consistent or peculiar traits.

**FIGURE 2:**
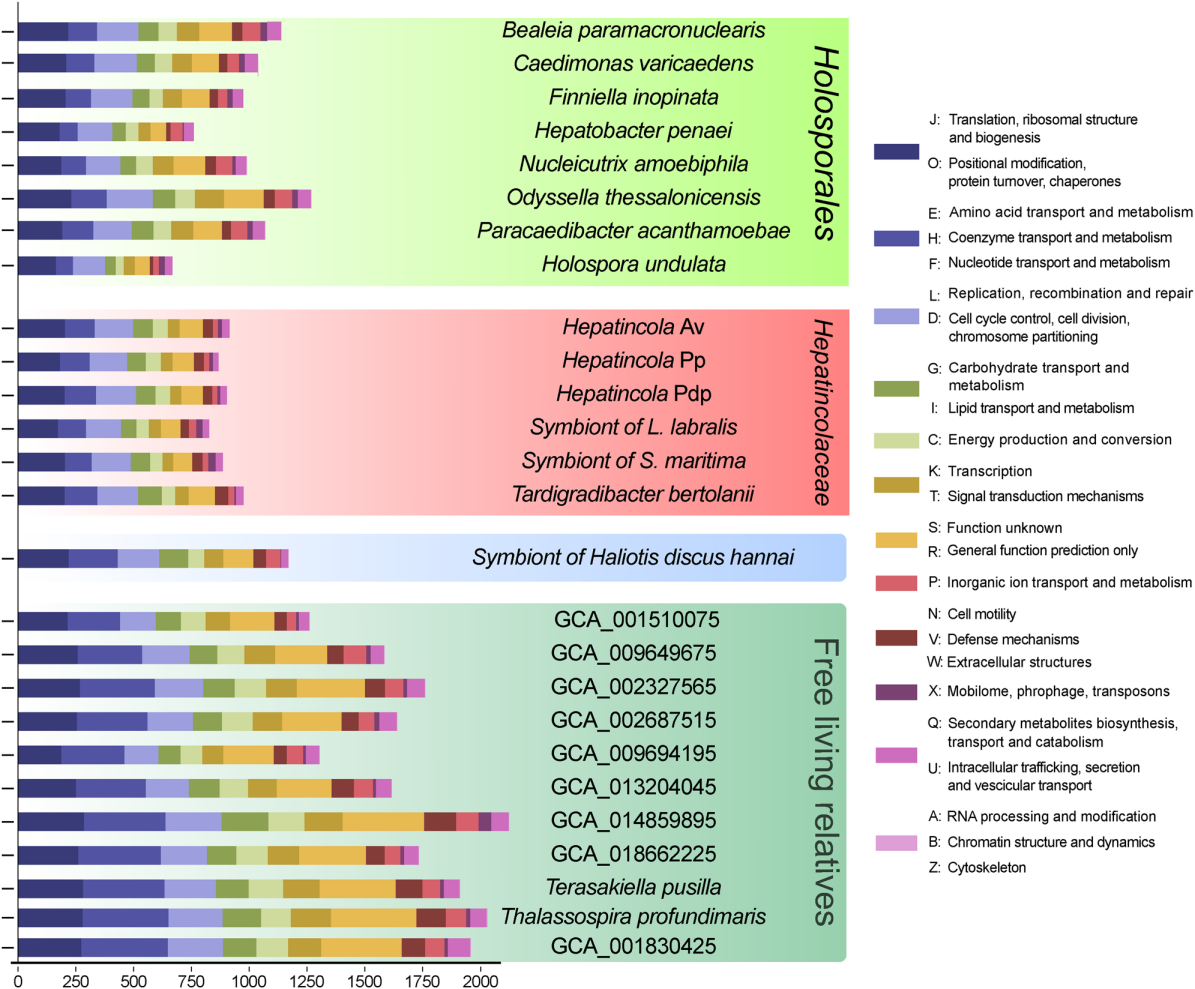
Barplot comparing the functional repertoire in terms of clusters of orthologous groups (COGs) of the *Hepatincolaceae* and their relatives. For viewers' clarity, categories were merged into loosely related groups. Bacteria are organised into four groups by their phylogeny and lifestyle, namely the *Holosporales* (light green background), the *Hepatincolaceae* (red background), the symbiont of *Haliotis* (blue background), and the free‐living relatives of the latter and the *Hepatincolaceae* (dark green background). *L. labralis* is an abbreviation for *Labiotermes labralis* and *S. maritima* for *Strigamia maritima*.

In terms of annotated COGs, the size of the functional repertoire of the *Hepatincolaceae* is quite homogeneous and within the typical range of the *Holosporales* (Figures 2 and S6; Table S10). However, it is not as large as one of the symbionts of *Haliotis* (which is consistent with the largest sets among *Holosporales*) or of their other close relatives, which, on average, have roughly twice as large COG repertoires (Figure 2). According to their remarkably larger COG sets, all the latter marine bacteria, including MAGs, were deemed as putatively non‐host‐dependent and will be referred to as ‘free‐living’ from now on. On the other hand, all the analysed herein *Holosporales*, including those with the largest genomes and COG repertoires, belong to well‐characterised host‐associated bacteria and consistently present a reduction in biosynthetic pathways (Categories E, F and H in Figure 2), comparably to the *Hepatincolaceae* (for a recent in‐depth analysis, see Giovannini, Petroni, and Castelli 2024).

**FIGURE 3:**
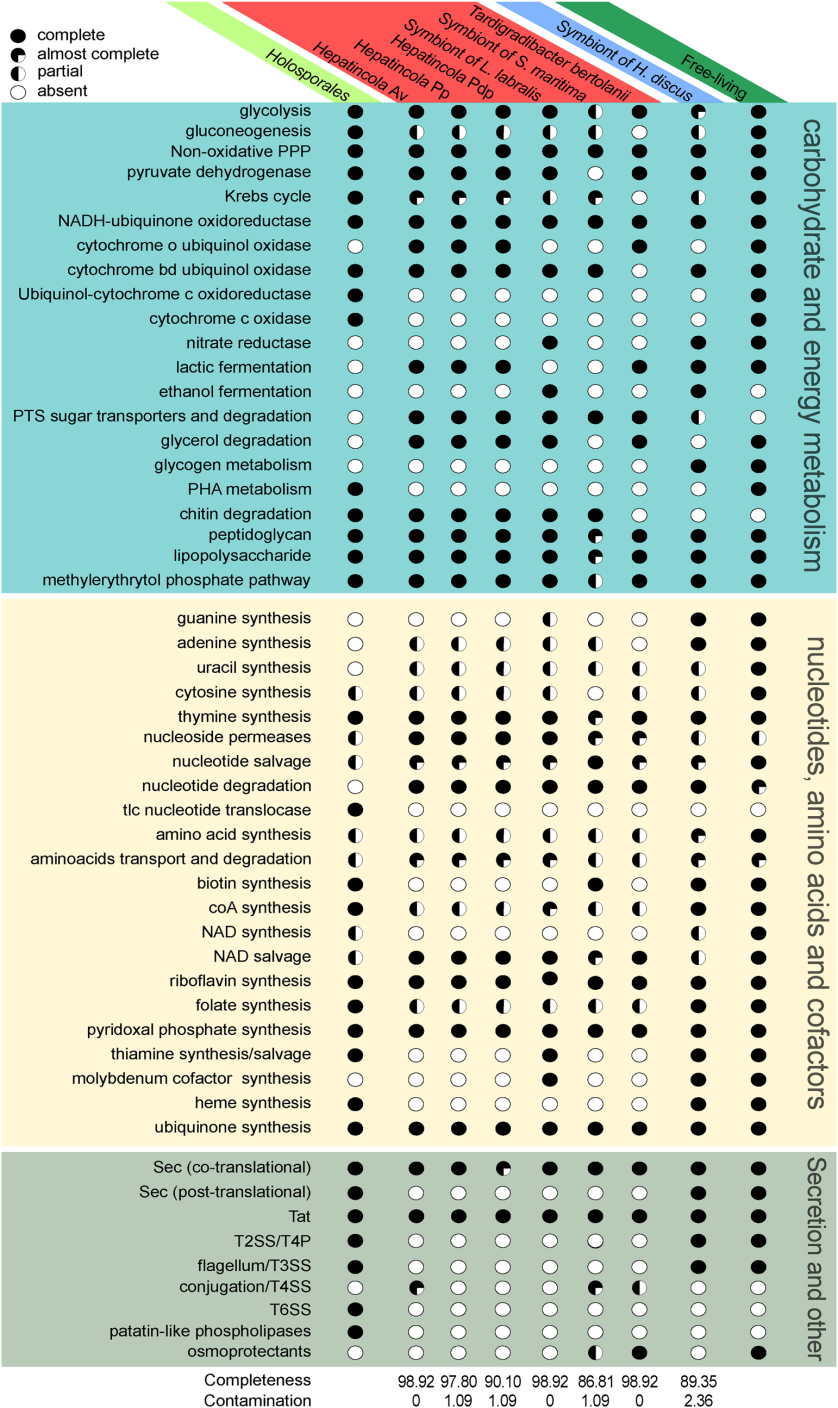
Graphical representation of the presence and completeness degree of selected metabolic pathways and functions in the *Hepatincolaceae* and the other bacteria analysed herein (*Holosporales*, symbiont of *Haliotis*, free‐living relatives). Pathways/functions are grouped by the coloured background in three broad sets, namely ‘carbohydrate and energy metabolism’ (teal), ‘nucleotides, amino acids and cofactors’ (yellow), and ‘secretion and other’ (olive green). The filling degree of each circle indicates the completeness of the corresponding function in the organism examined, namely complete (fully coloured), almost complete (three‐quarters coloured), partial (half coloured), absent (empty). For the *Holosporales* and the free‐living relatives, each circle stands for the completeness of the respective richest representative (for detailed information, see Supplementary Table S10 and Text S1). For reference, the completeness and contamination values of the individual genome assemblies according to CheckM (Table S7) are reported at the bottom lines.

We thus performed further, more detailed comparisons of the functional repertoires of the analysed organisms, focusing on differences among *Hepatincolaceae* and between *Hepatincolaceae* as a whole and the other investigated bacteria. Overall, the repertoires of the *Hepatincolaceae* and the other symbiotic bacteria investigated are largely subsets of one of their free‐living relatives, each with several lineage‐specific features (Figure S6). Such comparisons are summarised in Figure 3 and described in detail in (Text S1), with the main matters presented below and the corresponding COG repertoires reported in (Table S10).

### 3.4 Carbohydrate and Energy Metabolism

*Hepatincolaceae* can perform most of the core reactions in carbohydrate and energy metabolism (Figure 3; Table S10), as previously shown in *Hepatincola* spp. (Dittmer et al. 2023). They are capable of performing glycolysis, but can exert only a few steps that are specific to gluconeogenesis with respect to glycolysis (and are shared with the Krebs cycle; see Text S1 for details). Consistent with the symbiont of *Haliotis* and many *Holosporales*, the *Hepatincolaceae* present variable levels of reduction of the Krebs cycle, up to full absence in *Tardigradibacter*, and at least a minimal oxidative phosphorylation, which in some representatives includes the cytochrome o oxidase, absent in the other symbionts investigated, but is always devoid of cytochrome c reductase/oxidase, unlike many *Holosporales* (Figure 3).

Differently from the *Holosporales*, most *Hepatincolaceae* are also capable of getting energy in anaerobic conditions by either lactic or ethanol fermentation, the latter coupled with the ability to employ nitrate as a terminal acceptor (Figure 3).

The *Hepatincolaceae* are also equipped with a quite rich set of transporters that may enable them to obtain several metabolites from their hosts (Figure 3; see also Text S1). In particular, as previously shown in *Hepatincola* spp. (Dittmer et al. 2023), and unlike the free‐living relatives or the *Holosporales*, they have several phospho‐transferase system (PTS) sugar transporters.

Moreover, all the arthropod‐associated *Hepatincolaceae* (i.e., all except *Tardigradibacter*) can metabolise chitin or its derivatives, thanks to diverse enzymes, including chitinases (see Text S1 for details).

### 3.5 Metabolism of Nucleotides, Amino Acids and Cofactors

The nucleotide and amino acid synthesis abilities of the *Hepatincolaceae* are quite low (Figure 3; Table S10), as previously shown in *Hepatincola* spp. (Dittmer et al. 2023). Specifically, those for nucleotides are intermediate between the symbiont of *Haliotis* and the *Holosporales*. The *Hepatincolaceae* can also get deoxyribonucleotides via aerobic and, only in the symbiont of *Labiotermes*, anaerobic ribonucleotide reductases. Biosynthetic abilities for amino acids are even scarcer than those for nucleotides, similar to the average *Holosporales*, while the symbiont of *Haliotis* can produce almost all amino acids except for proline and tyrosine (Text S1).

Consistent with their poor biosynthetic capabilities, the *Hepatincolaceae* have several transporters for nucleotides, nucleosides and amino acids (Figure 3), which, however, do not include tlc nucleotide translocases, typical in *Holosporales* and other host‐associated intracellular bacteria (Major, Embley, and Williams 2017; Castelli et al. 2024; Mies et al. 2024).

The *Hepatincolaceae* also present versatile capabilities of purine salvage, and for degrading imported (deoxy)nucleosides and amino acids for energy production, with lineage‐specific patterns and partly shared traits with the other bacteria here analysed (Figure 3; Text S1).

On the other hand, all the *Hepatincolaceae* can perform *de novo* synthesis or salvage of several cofactors, either from initial precursors or from intermediates (Figure 3; Table S10; Text S1). Some other capabilities are found in a single representative, namely the symbiont of *Strigamia*, able to synthesise riboflavin and biotin, and the symbiont of *Labiotermes*, able to salvage thiamine diphosphate and synthesise the molybdenum cofactor, the latter being consistent with the presence of the molybdoenzyme nitrate reductase in the same bacterium, just like in the symbiont of *Haliotis* and many free‐living relatives.

### 3.6 Other Genomic Features

The *Hepatincolaceae* possess components of the Sec core translocon and co‐translational transport (Figure 3; Table S10). Regarding post‐translational transport, they lack the dedicated Sec component, but they do possess the Sec‐independent Tat secretion system. However, they do not present a number of secretion/interaction apparatuses that are typical of the *Holosporales* (Figure 3). At the same time, differently from all of the other bacteria analysed herein, *Hepatincola* Av, *Tardigradibacter* and the symbiont of *Strigamia* possess components of the Type IV secretion system, including the virD2 relaxase (Text S1).

We could also identify some protein classes that have been previously indicated as potentially involved in bacterial–host interactions (Text S1), with several lineage‐specific variations, in particular as compared to the *Holosporales*. However, *Hepatincolaceae*, the symbiont of *Haliotis* and their free‐living relatives do not have any patatin‐like phospholipase (Figure 3).

As most other bacteria herein analysed, all the *Hepatincolaceae* can produce the main components of the typical Gram‐negative cell membranes and walls, namely lipids, phospholipids, peptidoglycan, and lipopolysaccharide (Table S10; Text S1). Moreover, they all can perform mismatch repair, nucleotide excision repair, base‐excision repair, as well as single‐ and double‐strand break homologous recombination, consistent with the symbiont of *Haliotis* and most *Holosporales* (Text S1).

Interestingly, *Tardigradibacter* can synthesise the osmoprotectant glycine betaine from choline (Nau‐Wagner et al. 2012). Neither gene of this pathway was found in the other *Hepatincolaceae* (besides a ~30% truncated *betA* in symbiont of *Strigamia*) or the other symbionts investigated (Figure 3).

Most *Hepatincolaceae* possess several phage genes. In *Tardigradibacter*, these are grouped in two putative complete prophages, with high reciprocal sequence identity, each encoding multiple phage components. Many homologues of those genes could be found in the other *Hepatincolaceae*, in particular in the ‘prophage regions’ 1 (in *Hepatincola* Av and Pdp) and 3 (in all *Hepatincola* spp.) (Dittmer et al. 2023) as well as in the symbiont of *Strigamia*. The reciprocal sequence identities of phage genes among the *Hepatincolaceae* are higher than those of other bacterial lineages, suggestive of a common origin. However, the lack of synteny is also indicative of a non‐negligible evolutionary distance (Figure S7). Curiously, a prophage of *Tardigradibacter* encodes for two Type II toxin‐antitoxin systems. For only one of the antitoxins, we could detect a homologue among the *Hepatincolaceae*, namely in the symbiont of *Labiotermes*.

## 4 Discussion

The *Hepatincolaceae* (or ‘*Ca*. Tenuibacteraceae’; Kroer et al. 2016) are a lineage of *Alphaproteobacteria* living in the gut of ecdysozoan hosts, extracellularly associated with microvilli (Kroer et al. 2016; Dittmer et al. 2023). They were until now considered a sublineage of the *Holosporales* (Szokoli et al. 2016; Guidetti et al. 2020), which otherwise include only obligatorily intracellular bacteria prevalently hosted by protists (Giovannini, Petroni, and Castelli 2024). However, due to the paucity of genome sequences, such reconstructions were chiefly based on 16S rRNA gene sequences, which, due to a limited number of sites and compositional biases (low GC content), can be prone to artefacts and in analogous circumstances had led to incorrect grouping of phylogenetically independent host‐associated alphaproteobacterial lineages (e.g., *Rickettsiales* and *Holosporales*) (Ferla et al. 2013; Muñoz‐Gómez et al. 2019; Huang et al. 2020).

Herein, we took advantage of an extended genomic dataset of *Hepatincolaceae* and of methods to attenuate compositional biases (Muñoz‐Gómez et al. 2019) to reassess the phylogenetic affiliation of this lineage, showing that the grouping with the *Holosporales* was artefactual (Figure 1). According to our improved reconstruction, the *Hepatincolaceae* branched within the *Rhodospirillales* sensu Hördt et al. (2020), close to free‐living marine bacteria such as *Terasakiella* and *Thalassospira*, as well as to the symbiont of the mollusc *H. discus hannai* (Huang et al. 2020), which however branched within free‐living relatives independently from the *Hepatincolaceae*. Interestingly, and consistent with previous studies (Muñoz‐Gómez et al. 2019; Castelli et al. 2024), the *Holosporales* branched within the *Rhodospirillales* as well, but apart from the *Hepatincolaceae*, namely close to the families *Kiloniellaceae*, *Rhodovibrionaceae*, *Thalassobaculaceae* and *Azospirillaceae* (Figures 1 and S4). The actual phylogenetic proximity of the *Hepatincolaceae* and the *Holosporales* may have contributed to strengthening their artefactual grouping.

The *Hepatincolaceae* phylogeny suggests that they are the descendants of bacteria that ancestrally engaged in an association with a marine arthropod (or another ecdysozoan), possibly after being accidentally ingested, resisting digestion and later adapting to thrive therein. This seems different from the origin of the *Holosporales*, which has been tentatively linked to freshwater environments (Lanzoni et al. 2024). Such reconstruction of the origin of the *Hepatincolaceae* is also consistent with the fact that several representatives, including early diverging ones, were found in association with marine hosts (Kroer et al. 2016; Dittmer et al. 2023). The colonisation of hosts inhabiting different environments, such as freshwater and terrestrial, could have been favoured by associations with versatile hosts, such as certain crabs (Li et al. 2007), able to survive in both freshwater and seawater (Dittel and Epifanio 2009). Given their extracellular location, the *Hepatincolaceae* are most likely transmitted from host to host via a faecal–oral route (e.g., by egg‐smearing; Kikuchi et al. 2009) or by other feeding behaviours observed in known hosts, such as ingestion of exuviae (Tong et al. 2023) or carnivory (Lewis 1961). These mechanisms lead to an indirect vertical transmission, but may also favour horizontal transmission, including host species shift. Considering the inability to produce carbon storage forms such as glycogen or polyhydroxyalkanoate granules (Figure 3), differently from respectively the symbiont of *Haliotis* and several *Holosporales*, the *Hepatincolaceae* are probably engaged only in quite fast stages of passive transmission, a hypothesis also supported by the lack of flagellum (Figure 3). However, considering that all the *Hepatincolaceae* with sequenced genomes are associated with terrestrial hosts, as well as the environment‐dependent presence of flagella in other host‐associated alphaproteobacterial lineages (Castelli et al. 2024), it would be interesting to check whether aquatic ones bear flagella instead.

According to their genome features, the *Hepatincolaceae* present typical traits of obligatorily host‐associated bacteria, in particular, small genome size and absence of essential metabolic pathways, clearly indicative of marked metabolic dependence on their hosts. These features bring them together with the *Holosporales* and the closely related symbiont of *Haliotis*, representing cases of evolutionary convergence. However, significant specificities could be found in the *Hepatincolaceae* as a whole, which, besides being lineage‐specific traits, may also shed light on differences in lifestyle between these lineages (Figure S8). For example, we found that all the *Hepatincolaceae* display complex and robust surface structures (peptidoglycan and lipopolysaccharide), differently from some *Holosporales* (George et al. 2020; Castelli et al. 2022), as well as from representatives of the *Rickettsiales*, another group of intracellular *Alphaproteobacteria* (Lin and Rikihisa 2003; Min et al. 2008). They also lack patatin‐like phospholipases, which is implied in bacterial evasion from host intracellular vacuoles (Borgo et al. 2022) and is common among the *Holosporales*.

Relevant differences were also found in nutrient uptake and energy metabolism. The *Holosporales* live in quite stable environments, namely host cells, and thus can take advantage of constant metabolite supply. Accordingly, similarly to the *Rickettsiales* (Driscoll et al. 2017), their metabolism (and transporters) can accommodate and exploit a limited number of input molecules to be either consumed directly for ATP synthesis or converted to metabolic precursors, for example, by gluconeogenesis to get building blocks of peptidoglycan and lipopolysaccharide. On the other hand, the host gut where the *Hepatincolaceae* thrive, while potentially quite nutrient‐rich as well, is more variegated and variable, being characterised by undigested compounds from each different food source of the host, as well as their multiple intermediates of digestion. To fully exploit these conditions, it seems not surprising that the *Hepatincolaceae* are more flexible in terms of input molecules, bearing transporters and processing enzymes for multiple sugars and other carbon sources, including amino acids and nucleosides, as well as being able to either recycle or degrade them (Figure 3; Text S1). Conversely, the *Holosporales* mostly import nucleotides for biosynthesis purposes only. The presence of full glycolysis in the *Hepatincolaceae* seems consistent with this scenario (Figure 3), together with the significant reduction of gluconeogenesis, which is likely dispensable due to the adequate carbohydrate supply. It is also noteworthy that, while the *Holosporales* are strictly aerobic, the *Hepatincolaceae* have a quite reduced or even absent Krebs cycle, but, thanks to fermentation and/or alternative terminal electron acceptors, many of them can thrive in anaerobic conditions that can be encountered in host guts (Figure 3) (Engel and Moran 2013).

It is also interesting to observe that, besides basic systems such as Sec and Tat, the *Hepatincolaceae* are almost devoid of protein secretion systems, differently from the *Holosporales*, which typically have type II and type VI secretion systems (Figure 3), most probably implied in the interaction with host cells of these intracellular bacteria (George et al. 2020; Giovannini, Petroni, and Castelli 2024). The only partial exception could be the finding, only in some representatives of the *Hepatincolaceae*, of several components of the type IV secretion system (Figure 3), which may be enough for a functional secretory apparatus. At the same time, the presence of the relaxase component virD2 could indicate the role of this apparatus in DNA uptake and transfer (Christie 2016). Considering also the direct and indirect contact with diverse arrays of other bacteria in the host guts (Engel and Moran 2013; Schapheer, Pellens, and Scherson 2021), this may favour HGT events. Interestingly, we identified hints of potential HGTs in a number of cases, mostly involving genes related to the uptake and digestion of various nutrients, in particular multiple carbohydrates, including chitin, nucleosides, and amino acids (Table S11). This suggests a certain degree of genomic plasticity among the *Hepatincolaceae*, which may be related to the lineage‐specific adaptations among the *Hepatincolaceae* (see also below). In any case, putative effector molecules, in particular bearing ankyrin repeats, could be found in the *Hepatincolaceae* more frequently than in their free‐living relatives (Text S1), which is consistent with a possible modulation of host interactions.

Several highly related phage genes were found in most *Hepatincolaceae*, that is, in all except the symbiont of *Labiotermes*, although this assembly was published after metagenome binning (Hervé et al. 2020), and may thus lack accessory genomic components such as phages. These genes are probably functional, in particular in *Tardigradibacter*, where they are all organised in two putative complete prophages with high reciprocal identities (Figure S7). These findings are consistent with an ancestral presence and/or a lineage‐specific adaptation of such prophages in the *Hepatincolaceae*. Considering their conservation despite the quite reduced genomes, it is intriguing to wonder whether those prophages could exert some role in the lifestyle of *Hepatincolaceae* bacteria, particularly in interactions with the hosts. Along this line of thought, the presence of toxin‐antitoxin genes in one prophage of *Tardigradibacter* is noteworthy, being reminiscent of prophage‐linked toxins and antitoxins implied in the cytoplasmic incompatibility caused by *Wolbachia* in arthropod hosts (Beckmann et al. 2019).

Besides the common trends among the *Hepatincolaceae* presented above, some noteworthy lineage‐specific traits were identified, tentatively relatable to host‐dependent features. In particular, only *Tardigradibacter* can synthesise the osmoprotectant glycine betaine (Nau‐Wagner et al. 2012). This may enable the bacterium to survive during the cryptobiosis of its host (Møbjerg et al. 2011), in particular as a consequence of desiccation, and, in principle, may also contribute to the host's survival in such conditions. Other features of individual *Hepatincolaceae* bacteria can be linked to nutrient uptake in relation to different feeding behaviours of the hosts (Text S1). These include the capability to metabolise derivatives of plant pectins (Renard, Crépeau, and Thibault 1999) by the symbiont of *Labiotermes*, the import of chitobiose by the same bacterium, and, in general, the potential to metabolise chitin by all the arthropod‐associated *Hepatincolaceae*. These may involve the digestion of exoskeleton from ingested exuviae or preys (Lewis 1961; Tong et al. 2023), or, at least in termites, of the fungal cell wall (Marynowska et al. 2023). This diversity (including likely non‐orthologous genes for chitin degradation) and other variations (Text S1) are also consistent with a possible role exerted by potential HGT events in the evolutionary adaptations of the *Hepatincolaceae* (Table S11).

With regard to the symbiont of *Haliotis*, although it has been observed as an intracellular bacterium (Horwitz, Mouton, and Coyne 2016), it shares many genomic traits with the *Hepatincolaceae* rather than with the *Holosporales*. These include anaerobic metabolism, capability to salvage nucleotides and their degradation for energy supply, biosynthetic capabilities for nucleotides and amino acids (the latter much richer than the *Hepatincolaceae*), and lack of secretion systems or patatin‐like phospholipases (Figure 3; Text S1). This resemblance might even go beyond the high phylogenetic proximity, hinting at a more complex life cycle of this bacterium.

In summary, our study provides evidence that the phylogenetic grouping of *Hepatincolaceae* with the *Holosporales* was artefactual, similar to other known cases among host‐associated and AT‐rich *Alphaproteobacteria* (Martijn et al. 2018; Muñoz‐Gómez et al. 2019), thereby suggesting that such artefacts may be more common than currently recognised. Our findings allow us to re‐think the features of the *Hepatincolaceae* from a novel evolutionary perspective. In particular, thanks also to the significantly increased number of genome sequences made herein available, we identified multiple traits involved in their tailored adaptation to life inside the gut of arthropods and other ecdysozoans. Besides still conjectural indications of a possible contribution of *Tardigradibacter* to host cryptobiosis, our data overall confirm previous indications that the *Hepatincolaceae* most likely behave as nutrient scavengers of gut nutrients rather than being beneficial for their hosts (Dittmer et al. 2023). This parasitic behaviour brings them close to the actual *Holosporales* (Garushyants et al. 2018; George et al. 2020; Castelli et al. 2022; Giovannini, Petroni, and Castelli 2024), which, given the updated phylogenetic scenario, however represents a case of convergent evolution, just like the shared traits among *Holosporales* and *Rickettsiales* (Muñoz‐Gómez et al. 2019). Further genomic studies with extended datasets, as well as direct experimental investigations, will be necessary to shed further light on the evolution of the *Hepatincolaceae* and their interactions with their hosts.

### 4.1 Description of ‘*Candidatus* Tardigradibacter bertolanii’ gen. nov. sp. nov.

‘*Candidatus* Tar.di.gra.di.bac'ter ber.to.la'ni.i’ (N.L. masc. n. *Tardigradibacter*, ‘tardus’, slow, ‘gradus’, pace, gait, and ‘bacter’, rod; N.L. adj. *bertolanii*, in honour of Professor emeritus Roberto Bertolani).

A bacterium found in association with the tardigrade *R*. cf. *coronifer*, originating from the moss in Öland, Sweden. According to genome features, devoid of flagella or pili, and capable of both aerobic and anaerobic growth. It may survive during host cryptobiosis. Basis of assignment: SSU rRNA gene sequence (accession number: MK028537) and complete genome sequence (accession number: JBEULC000000000).

## Author Contributions

**Michele Castelli:** conceptualization, investigation, methodology, formal analysis, supervision, data curation, writing – original draft. **Leandro Gammuto:** methodology, data curation, investigation, formal analysis, visualization. **Diona Podushkina:** investigation, methodology. **Matteo Vecchi:** resources, visualization, writing – review and editing. **Tiziana Altiero:** writing – review and editing, resources. **Emanuela Clementi:** writing – review and editing, methodology. **Roberto Guidetti:** writing – review and editing, investigation, resources. **Lorena Rebecchi:** resources, writing – review and editing, investigation. **Davide Sassera:** writing – review and editing, funding acquisition, conceptualization, resources, validation.

## Conflicts of Interest

The authors declare no conflicts of interest.

## Supporting information


**Figure S1.** Plot of the contigs of the preliminary assembly of *Richtersius* cf. *coronifer* that bears ‘*Candidatus* Tardigradibacter bertolanii’. Contigs are shown according to their GC content and log 10 of sequencing coverage, and coloured according to the respective best megablast hit. Only contigs with length higher than or equal to 1000 bp are shown for viewers’ clarity.

**Figure S2.** Plot of the contigs of the preliminary assembly of *Strigamia maritima* bearing a ‘*Candidatus* Hepatincolaceae’ symbiont. Contigs are shown according to their GC content and log 10 of sequencing coverage, and coloured according to the respective best DIAMOND blastx hit. Only contigs with length higher than or equal to 1000 bp are shown for viewers’ clarity.

**Figure S3.** Graphical representation of the final genome assembly of ‘*Candidatus* Tardigradibacter bertolanii’, including connections between contigs based on the SPAdes assembly graph, obtained with the Bandage software (https://doi.org/10.1093/bioinformatics/btv383).

**Figure S4.** Complete maximum likelihood phylogenomic trees of the *Hepatincolaceae* and other *Alphaproteobacteria*. The presented trees are, in the order, the one inferred on the untreated concatenated alignment, and on those with 10%, 20%, 30%, 40%, and 50% most compositionally biassed sites removed. The novel *Tardigradibacter bertolanii* is highlighted in red. Numbers on branches stand for support values by SH‐aLRT with 1000 replicates and by 1000 ultra‐fast bootstraps (full support values were omitted for readers’ clarity). The tree scale stands for estimated proportional sequence divergence.

**Figure S5.** Maximum likelihood phylogenetic tree on the SSU rRNA gene of *Hepatincolaceae* and their relatives. The novel *Tardigradibacter bertolanii* and the closely related Otu000635 are highlighted in red. Numbers on branches stand for boostrap supports after 1000 pseudo‐replicates (values below 70% were omitted). The scale bar stands for estimated proportional sequence divergence.

**Figure S6.** (a) Barplot comparing the functional repertoire in terms of clusters of orthologous groups (COGs) of the *Hepatincolaceae* and their relatives, as in Figure 2, but showing relative proportions. For viewers’ clarity, categories were merged into loosely related groups. Bacteria are organised in four groups by their phylogeny and lifestyle, namely the *Holosporales* (light green background), the *Hepatincolaceae* (red background), the symbiont of *Haliotis* (blue background), and the free‐living relatives of the latter and the *Hepatincolaceae* (dark green background). (b) Venn diagram comparison of the COG repertoires of those four groups. (c) Venn diagram comparison of COG repertoires among the *Hepatincolaceae*, namely *Tardigradibacter*, the members of genus *Hepatincola*, and the symbionts of *Strigamia* and *Labiotermes*.

**Figure S7.** Scheme of the genomic location of phage genes in the two putative prophage regions of *Tardigradibacter bertolanii* and their detected homologues in the other *Hepatincolaceae* (for the sake of brevity, only one *Hepatincola* is shown). Each line stands for a distinct assembled contig (except for *Hepatincola* Av, where only two genomic segmentsm corresponding to the prophage regions 1 and 3 by Dittmer et al. 2023, are shown), and each arrow indicates the direction of the respective gene, coloured according to the annotated function.

**Figure S8.** Graphic and textual summary of the main features distinguishing the ‘*Ca*. Hepatincolaceae’ and the *Holosporales*. The former are non‐motile bacteria thriving extracellularly in the host gut lumen, in association with microvilli, while the latter are intracellular bacteria, mostly hosted by protists such as amoebae and ciliates, and frequently bearing flagellar genes. Major differences in the respective genomic repertoires, consistent with such unlike lifestyles, are listed in the text boxes.

**Table S1.** List of the contigs of the preliminary assembly of *Richtersius coronifer* bearing ‘*Candidatus* Tardigradibacter bertolanii’ in which rRNA genes were identified with barrnap. For each contig, blobology parameters are reported, as well as the positions in which the rRNA gene was inferred, and the best blast hit of this gene sequence. Contigs are sorted by the respective sequencing coverage, and coloured according to the presumed organismal assignment (blue: host; shades of green: ‘*Candidatus* Tardigradibacter bertolanii’; red: additional bacteria).

**Table S2.** PCR primer combinations, sequences, and amplification protocols used for joining contigs of the Illumina genome assembly of ‘*Candidatus* Tardigradibacter bertolanii’.

**Table S3.** List of organism and respective accession numbers used for the phylogenomic analyses.

**Table S4.** Presence/absence and total number of the 179 eggNOGs used for phylogeny identified in the six ‘*Candidatus* Hepatincolaceae’ genome assemblies.

**Table S5.** Tardigrade microbiome samples analysed in this study for the presence of relative of ‘*Candidatus* Tardigradibacter bertolanii’, including the respective references and the link to the source were reads or precomputed OTUs were downloaded.

**Table S6.** Genome assembly statistics among the ‘*Candidatus* Hepatincolaceae’.

**Table S7.** Results of the CheckM analyses of the ‘*Candidatus* Hepatincolaceae’ genome assemblies and the other alphaproteobacterial MAGs included in the phylogenomic dataset, including in particular the ‘Completeness’ and ‘Contamination’ scores.

**Table S8.** ANI values among the ‘*Candidatus* Hepatincolaceae’ genome assemblies.

**Table S9.** SRA accession numbers of the tardigrade microbiome samples that included the OTU000635, identified as a close relative of ‘*Candidatus* Tardigradibacter bertolanii’.

**Table S10‐1.** List of COGs, including the respective descriptions and functional categories, for all the genomes included in the comparative analyses.

**Table S10‐2.** List of COGs, including the respective descriptions and functional categories, for all the genomes included in the comparative analyses.

**Table S11.** List of the genes of ‘*Candidatus* Hepatincolaceae’ bacteria involved in putative HGT events, including the representative(s) in which they were identified, and the reason why they were deemed as putatively transferred.

**Text S1.** Detailed description of the metabolic features of the ‘*Candidatus* Hepatincolaceae’ in comparison with host‐associated and free‐living relatives.

## Data Availability

Sequences from this study are available under NCBI BioProject PRJNA1112326: https://www.ncbi.nlm.nih.gov/bioproject/PRJNA1112326, including the assembly for *Tardigradibacter bertolanii* (symbiont of *Richtersius* cf. *coronifer*: JBEULC000000000) and symbionts of *Strigamia maritima* (JBEXBW000000000). All the Supporting Information files on Zenodo are now accessible under the following doi: 10.5281/zenodo.14286905.
