# Supplementary figures and images for: Hepatincolaceae (Alphaproteobacteria) are Distinct From Holosporales and Independently Evolved to Associate With Ecdysozoa
Source: Environ Microbiol. 2025 Jan 10;27(1):e70028. doi: 10.1111/1462-2920.70028 (PMC11724238; doi:10.1111/1462-2920.70028)

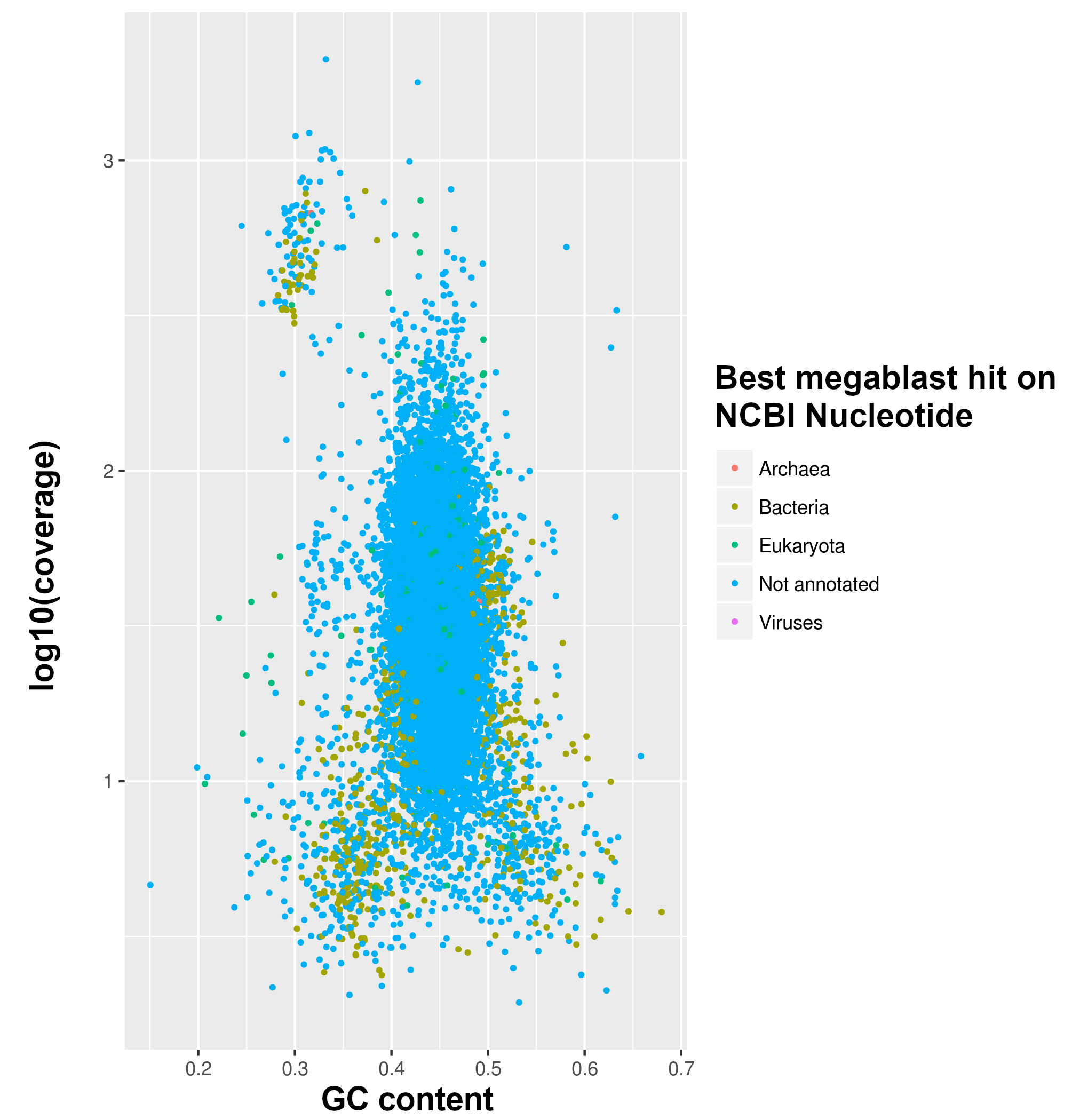

Supplement: Supplementary file 1 — Figure S1. Plot of the contigs of the preliminary assembly of Richtersius cf. coronifer that bears ‘Candidatus Tardigradibacter bertolanii’. Contigs are shown according to their GC content and log 10 of sequencing coverage, and coloured according to the respective best megablast hit. Only contigs with length higher than or equal to 1000 bp are shown for viewers’ clarity. [file EMI-27-e70028-s016.png]

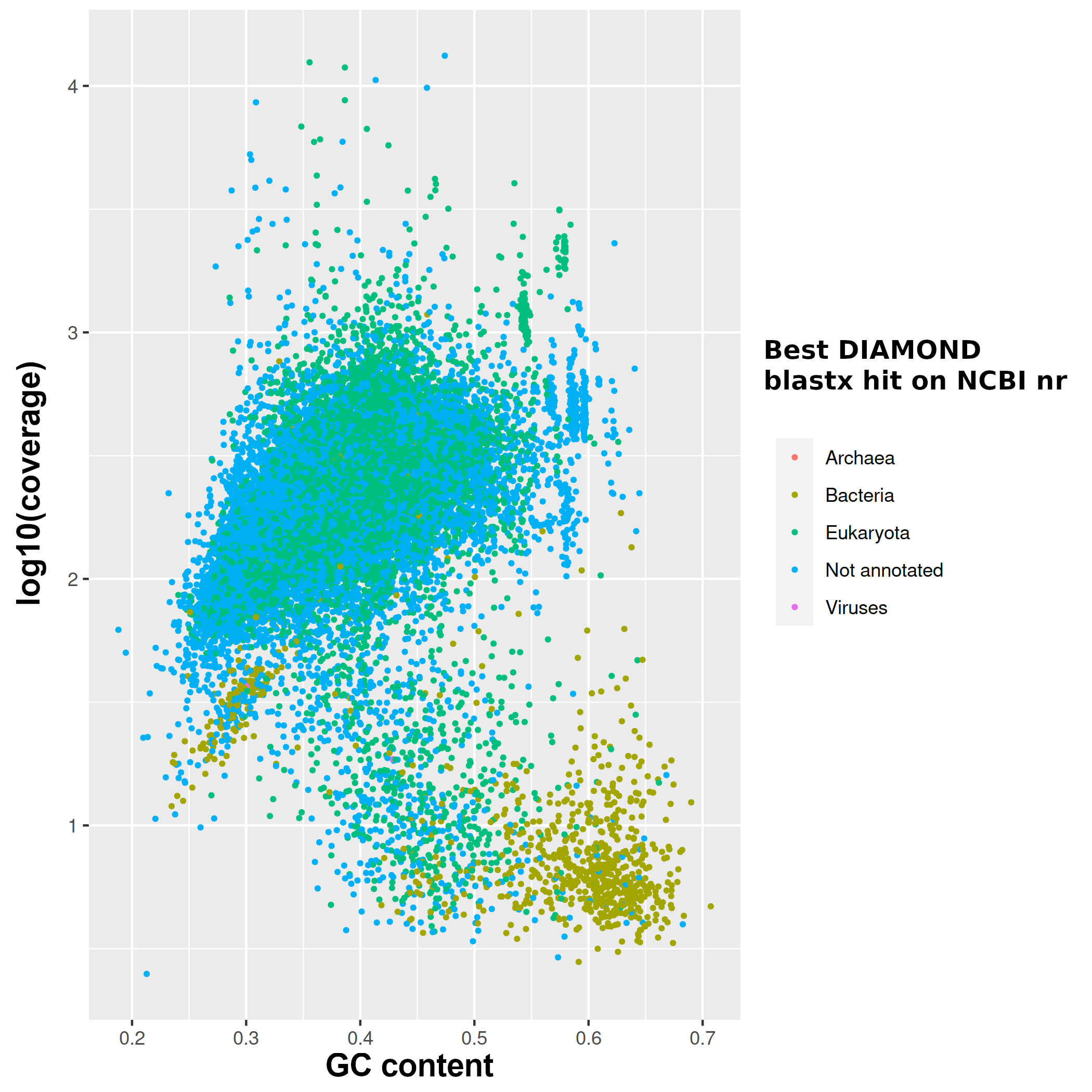

Supplement: Supplementary file 2 — Figure S2. Plot of the contigs of the preliminary assembly of Strigamia maritima bearing a ‘Candidatus Hepatincolaceae’ symbiont. Contigs are shown according to their GC content and log 10 of sequencing coverage, and coloured according to the respective best DIAMOND blastx hit. Only contigs with length higher than or equal to 1000 bp are shown for viewers’ clarity. [file EMI-27-e70028-s012.png]

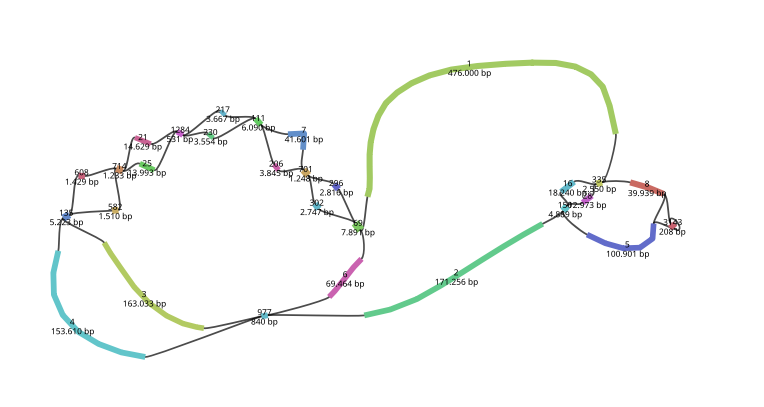

Supplement: Supplementary file 3 — Figure S3. Graphical representation of the final genome assembly of ‘Candidatus Tardigradibacter bertolanii’, including connections between contigs based on the SPAdes assembly graph, obtained with the Bandage software (https://doi.org/10.1093/bioinformatics/btv383). [file EMI-27-e70028-s002.png]

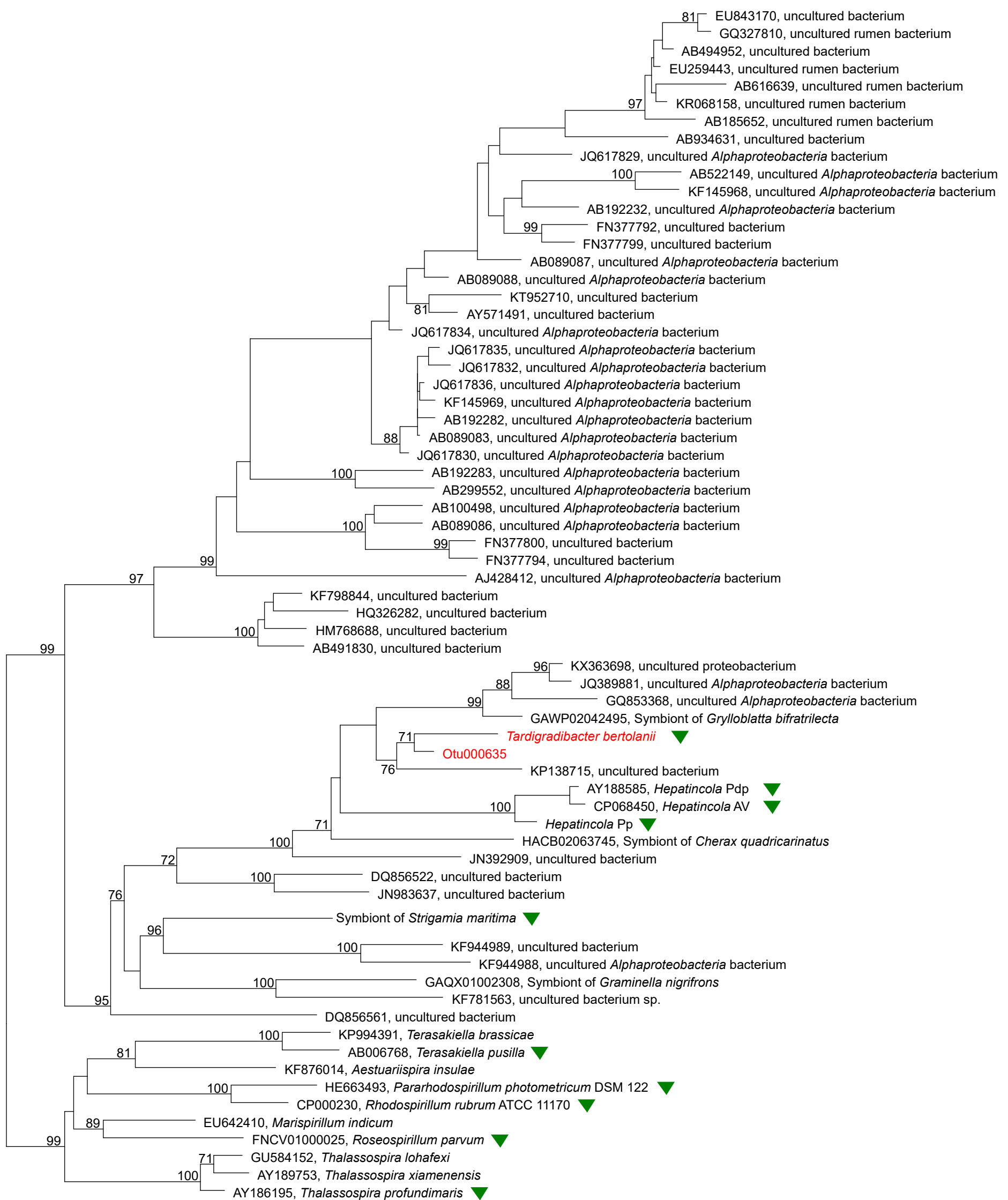

Supplement: Supplementary file 5 — Figure S5. Maximum likelihood phylogenetic tree on the SSU rRNA gene of Hepatincolaceae and their relatives. The novel Tardigradibacter bertolanii and the closely related Otu000635 are highlighted in red. Numbers on branches stand for boostrap supports after 1000 pseudo‐replicates (values below 70% were omitted). The scale bar stands for estimated proportional sequence divergence. [file EMI-27-e70028-s018.pdf]
