# Supplementary material for: Hepatincolaceae (Alphaproteobacteria) are Distinct From Holosporales and Independently Evolved to Associate With Ecdysozoa
Source: Environ Microbiol. 2025 Jan 10;27(1):e70028. doi: 10.1111/1462-2920.70028 (PMC11724238; doi:10.1111/1462-2920.70028)

Phylogenomic analyses - Original alignment

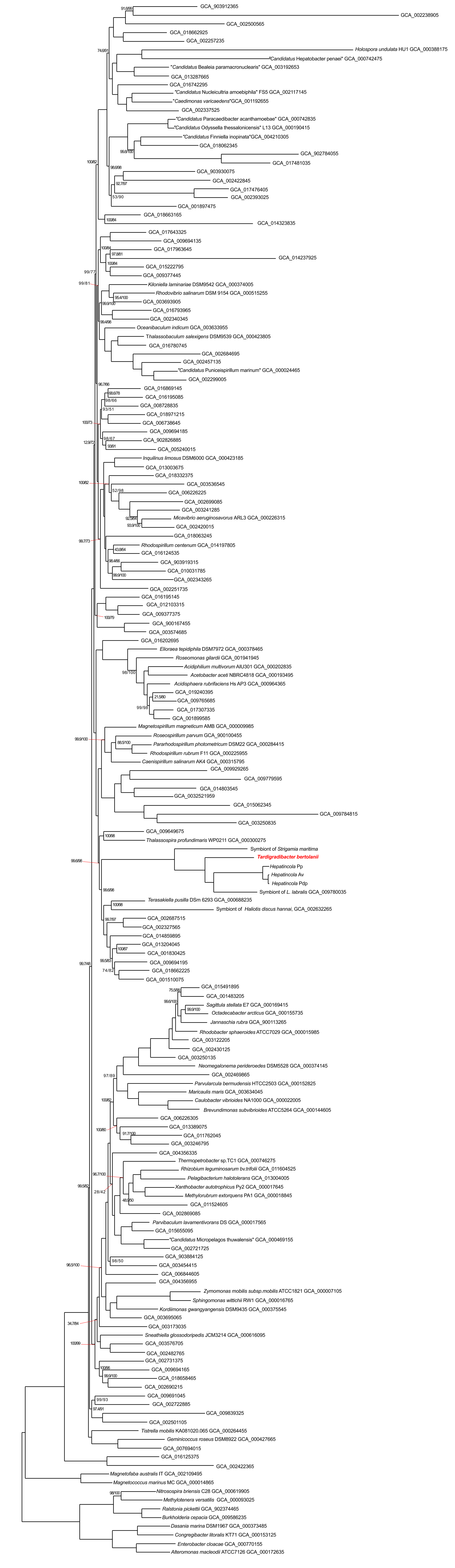

Phylogenomic analyses - 10% of most heterogenous sites removed

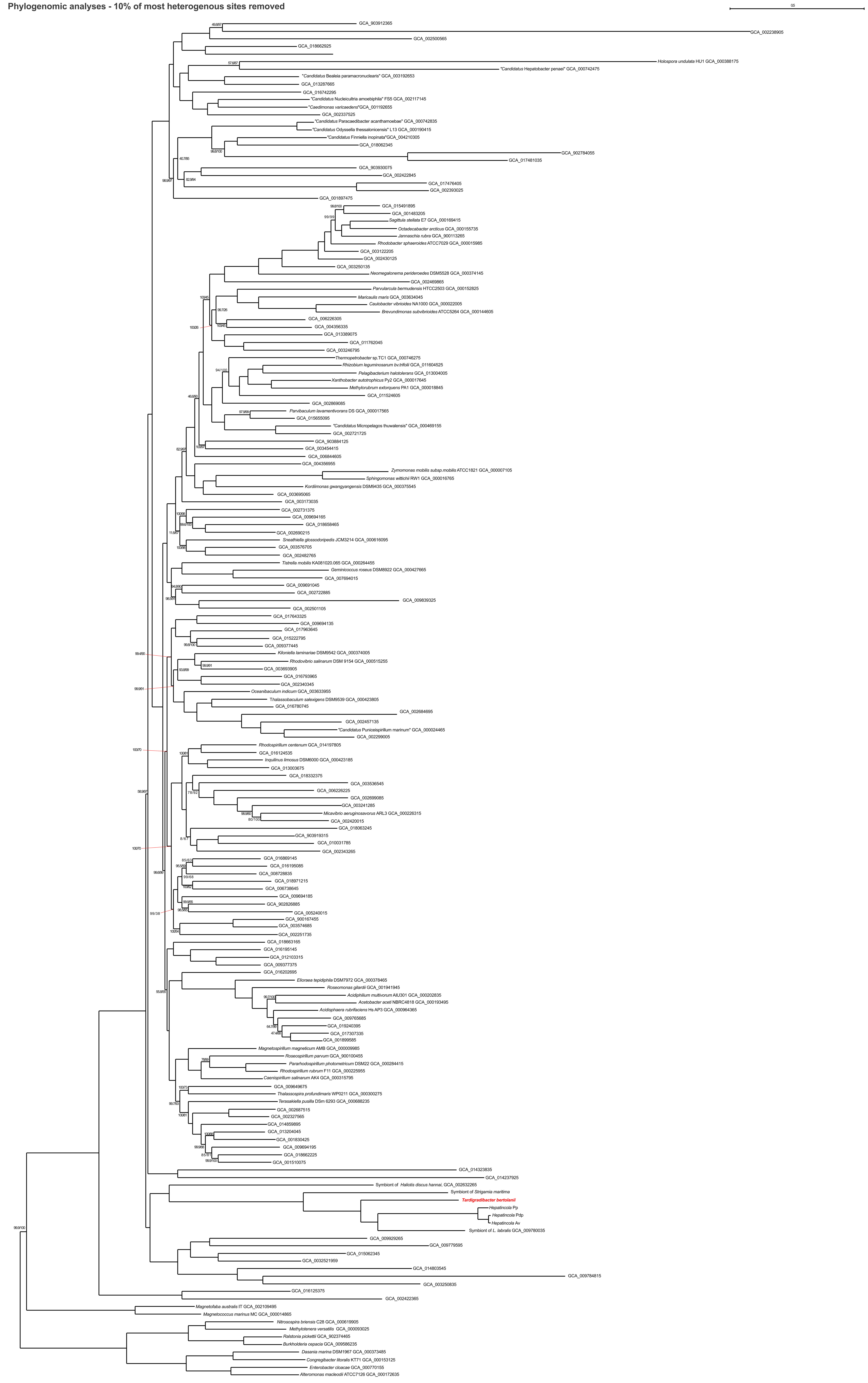

Phylogenomic analyses - 20% of most heterogenous sites removed

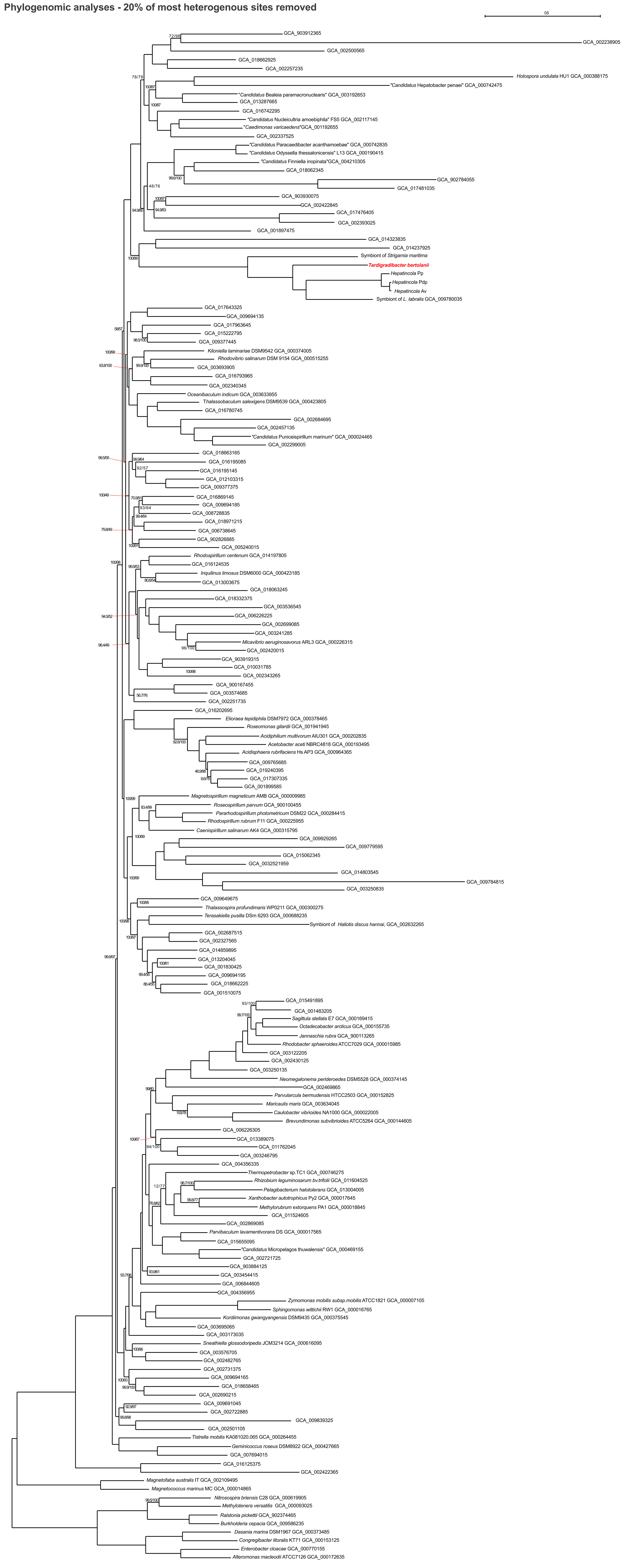

Phylogenomic analyses - 30% of most heterogenous sites removed

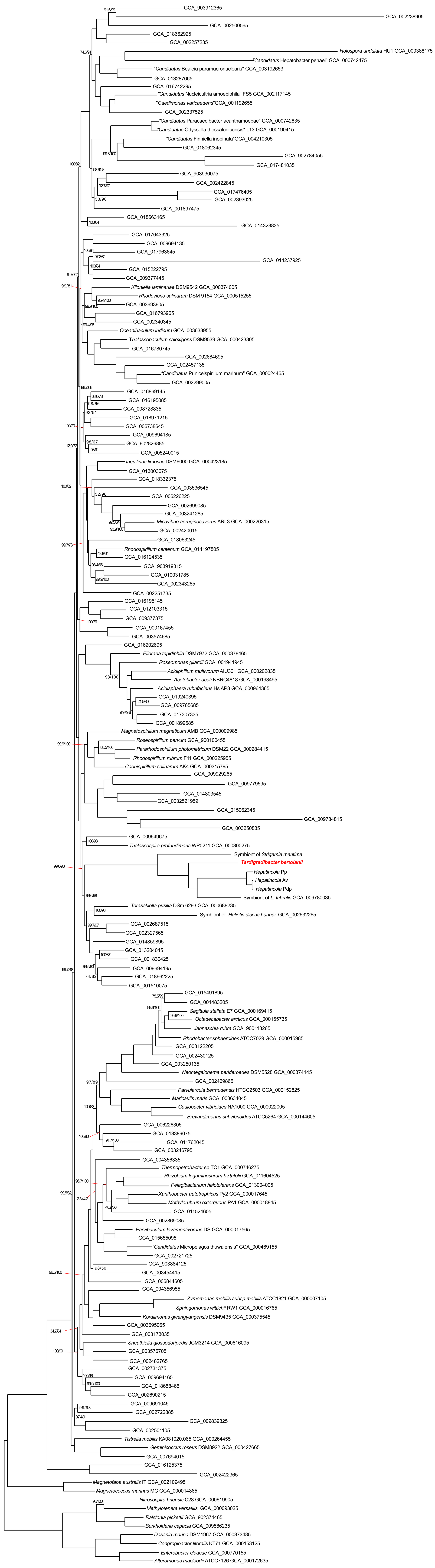

Phylogenomic analyses - 40% of most heterogenous sites removed

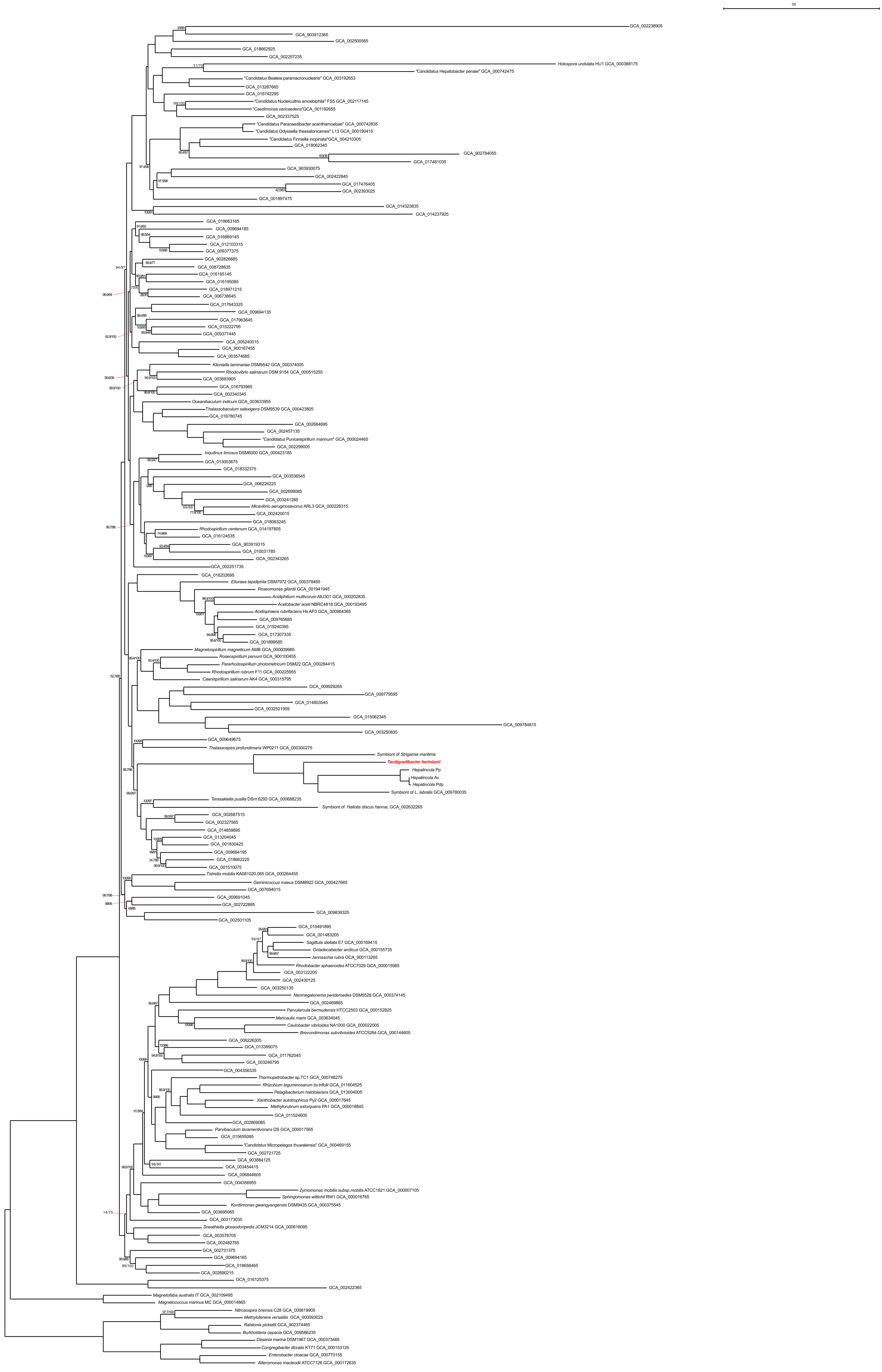

Phylogenomic analyses - 50% of most heterogenous sites removed

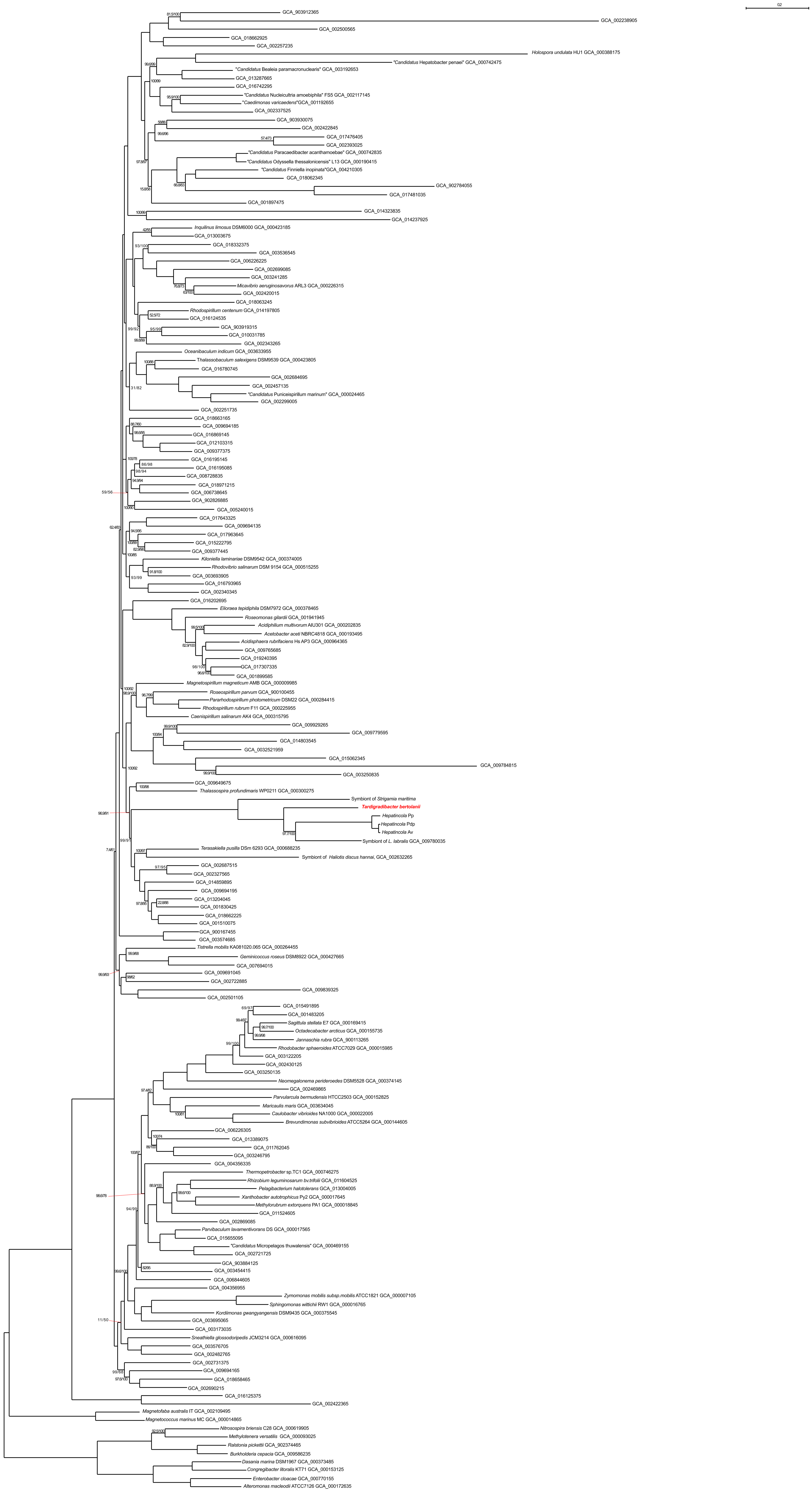

Supplement: Supplementary file 4 — Figure S4. Complete maximum likelihood phylogenomic trees of the Hepatincolaceae and other Alphaproteobacteria. The presented trees are, in the order, the one inferred on the untreated concatenated alignment, and on those with 10%, 20%, 30%, 40%, and 50% most compositionally biassed sites removed. The novel Tardigradibacter bertolanii is highlighted in red. Numbers on branches stand for support values by SH‐aLRT with 1000 replicates and by 1000 ultra‐fast bootstraps (full support values were omitted for readers’ clarity). The tree scale stands for estimated proportional sequence divergence. [file EMI-27-e70028-s011.pdf]
