# Supplementary material for: Hepatincolaceae (Alphaproteobacteria) are Distinct From Holosporales and Independently Evolved to Associate With Ecdysozoa
Source: Environ Microbiol. 2025 Jan 10;27(1):e70028. doi: 10.1111/1462-2920.70028 (PMC11724238; doi:10.1111/1462-2920.70028)
Supplement: Supplementary file 6 — Figure S6. (a) Barplot comparing the functional repertoire in terms of clusters of orthologous groups (COGs) of the Hepatincolaceae and their relatives, as in Figure 2, but showing relative proportions. For viewers’ clarity, categories were merged into loosely related groups. Bacteria are organised in four groups by their phylogeny and lifestyle, namely the Holosporales (light green background), the Hepatincolaceae (red background), the symbiont of Haliotis (blue background), and the free‐living relatives of the latter and the Hepatincolaceae (dark green background). (b) Venn diagram comparison of the COG repertoires of those four groups. (c) Venn diagram comparison of COG repertoires among the Hepatincolaceae, namely Tardigradibacter, the members of genus Hepatincola, and the symbionts of Strigamia and Labiotermes. [file EMI-27-e70028-s020.pdf]

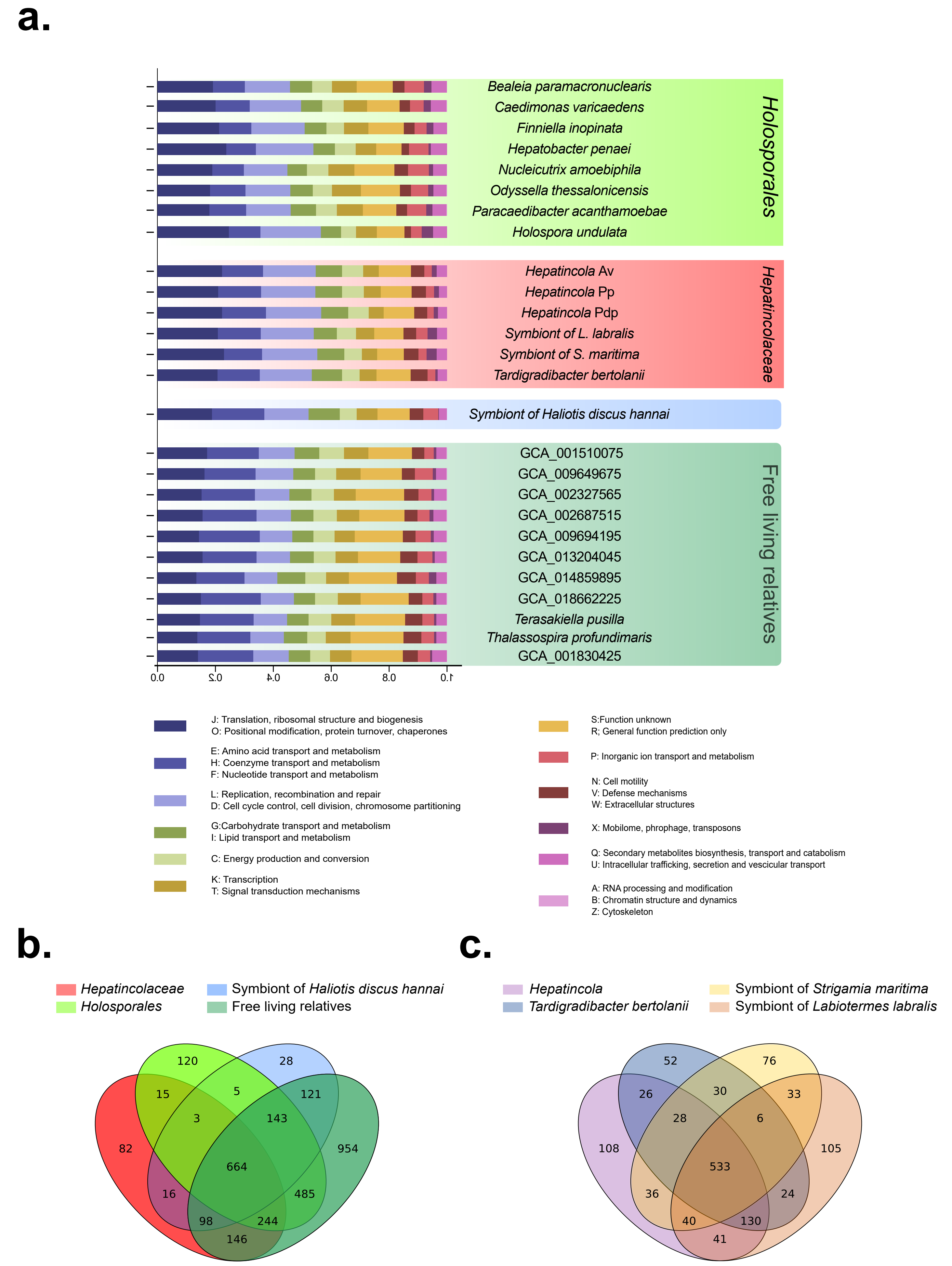

a.
Bealeia paramacronuclearis
Caedimonas varicaedens
Finniella inopinata
Hepatobacter penaei
Nucleicutrix amoebiphila
Odyssella thessalonicensis
Paracaedibacter acanthamoebae
Holospora undulata
Holosporales
Hepatincola Av
Hepatincola Pp
Hepatincola Pdp
Symbiont of L. labralis
Symbiont of S. maritima
Tardigradibacter bertolanii
Hepatincolaceae
Symbiont of Haliotis discus hannai
GCA_001510075
GCA_009649675
GCA_002327565
GCA_002687515
GCA_009694195
GCA_013204045
GCA_014859895
GCA_018662225
Terasakiella pusilla
Thalassospira profundimaris
GCA_001830425
Free living relatives
0.0
J: Translation, ribosomal structure and biogenesis
O: Positional modification, protein turnover, chaperones
E: Amino acid transport and metabolism
H: Coenzyme transport and metabolism
F: Nucleotide transport and metabolism
L: Replication, recombination and repair
D: Cell cycle control, cell division, chromosome partitioning
G:Carbohydrate transport and metabolism
I: Lipid transport and metabolism
C: Energy production and conversion
K: Transcription
T: Signal transduction mechanisms
S:Function unknown
R; General function prediction only
P: Inorganic ion transport and metabolism
N: Cell motility
V: Defense mechanisms
W: Extracellular structures
X: Mobilome, prophage, transposons
Q: Secondary metabolites biosynthesis, transport and catabolism
U: Intracellular trafficking, secretion and vescicular transport
A: RNA processing and modification
B: Chromatin structure and dynamics
Z: Cytoskeleton
b.
Hepatincolaceae
Holosporales
Symbiont of Haliotis discus hannai
Free living relatives
120
28
15
5
121
3
143
82
954
664
16
485
98
244
146
c.
Hepatincola
Tardigradibacter bertolanii
Symbiont of Strigamia maritima
Symbiont of Labiotermes labralis
52
76
26
30
33
28
6
108
105
533
36
24
40
130
41
