# Supplementary material for: Hepatincolaceae (Alphaproteobacteria) are Distinct From Holosporales and Independently Evolved to Associate With Ecdysozoa
Source: Environ Microbiol. 2025 Jan 10;27(1):e70028. doi: 10.1111/1462-2920.70028 (PMC11724238; doi:10.1111/1462-2920.70028)
Supplement: Supplementary file 7 — Figure S7. Scheme of the genomic location of phage genes in the two putative prophage regions of Tardigradibacter bertolanii and their detected homologues in the other Hepatincolaceae (for the sake of brevity, only one Hepatincola is shown). Each line stands for a distinct assembled contig (except for Hepatincola Av, where only two genomic segmentsm corresponding to the prophage regions 1 and 3 by Dittmer et al. 2023, are shown), and each arrow indicates the direction of the respective gene, coloured according to the annotated function. [file EMI-27-e70028-s005.pdf]

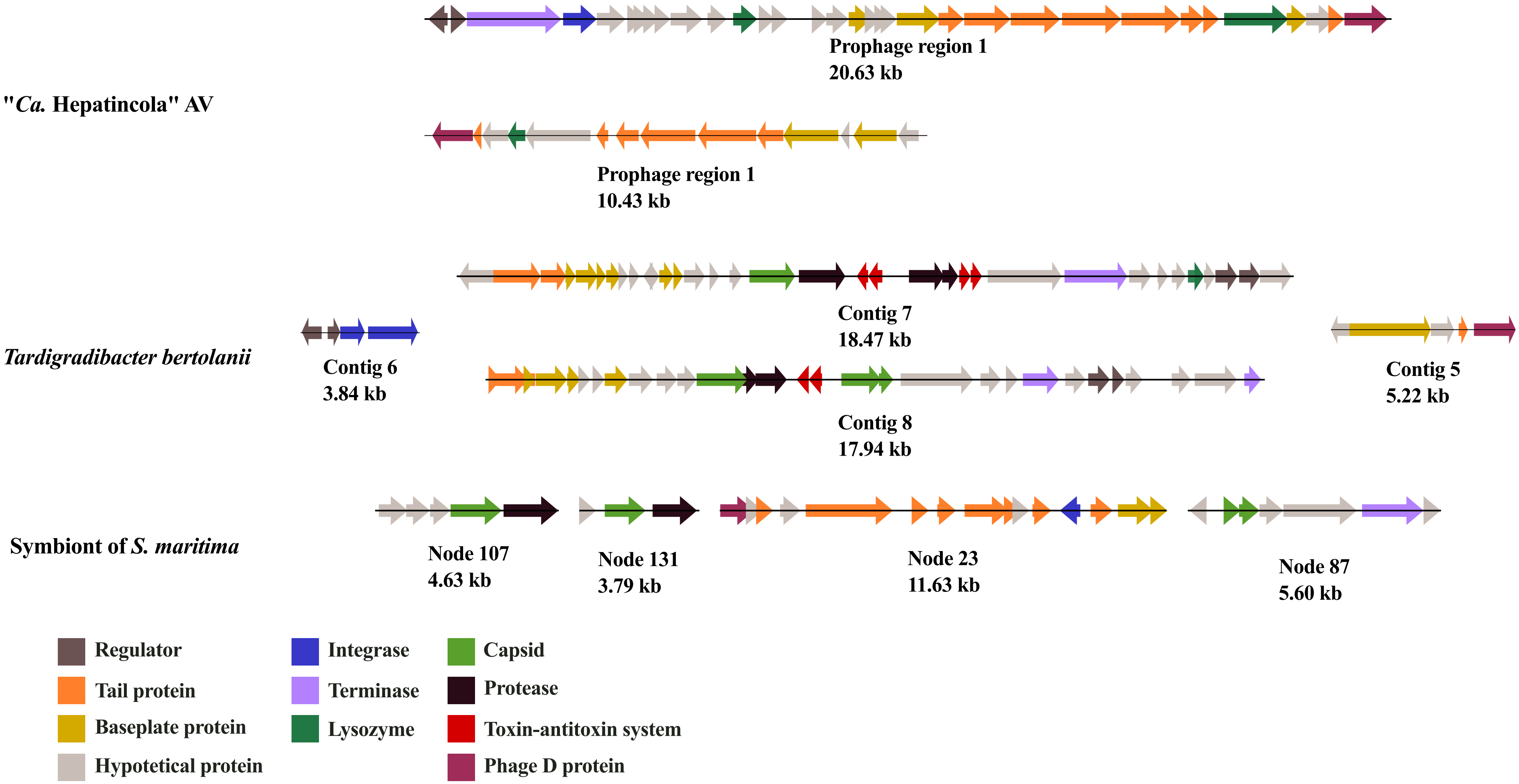

Prophage region 1
20.63 kb
"Ca. Hepatincola" AV
Prophage region 1
10.43 kb
Contig 7
18.47 kb
Tardigradibacter bertolanii
Contig 6
3.84 kb
Contig 5
5.22 kb
Contig 8
17.94 kb
Symbiont of S. maritima
Node 107
4.63 kb
Node 131
3.79 kb
Node 23
11.63 kb
Node 87
5.60 kb
Regulator
Tail protein
Baseplate protein
Hypotetical protein
Integrase
Terminase
Lysozyme
Capsid
Protease
Toxin-antitoxin system
Phage D protein
