# Supplementary material for: Hepatincolaceae (Alphaproteobacteria) are Distinct From Holosporales and Independently Evolved to Associate With Ecdysozoa
Source: Environ Microbiol. 2025 Jan 10;27(1):e70028. doi: 10.1111/1462-2920.70028 (PMC11724238; doi:10.1111/1462-2920.70028)
Supplement: Supplementary file 8 — Figure S8. Graphic and textual summary of the main features distinguishing the ‘Ca. Hepatincolaceae’ and the Holosporales. The former are non‐motile bacteria thriving extracellularly in the host gut lumen, in association with microvilli, while the latter are intracellular bacteria, mostly hosted by protists such as amoebae and ciliates, and frequently bearing flagellar genes. Major differences in the respective genomic repertoires, consistent with such unlike lifestyles, are listed in the text boxes. [file EMI-27-e70028-s013.pdf]

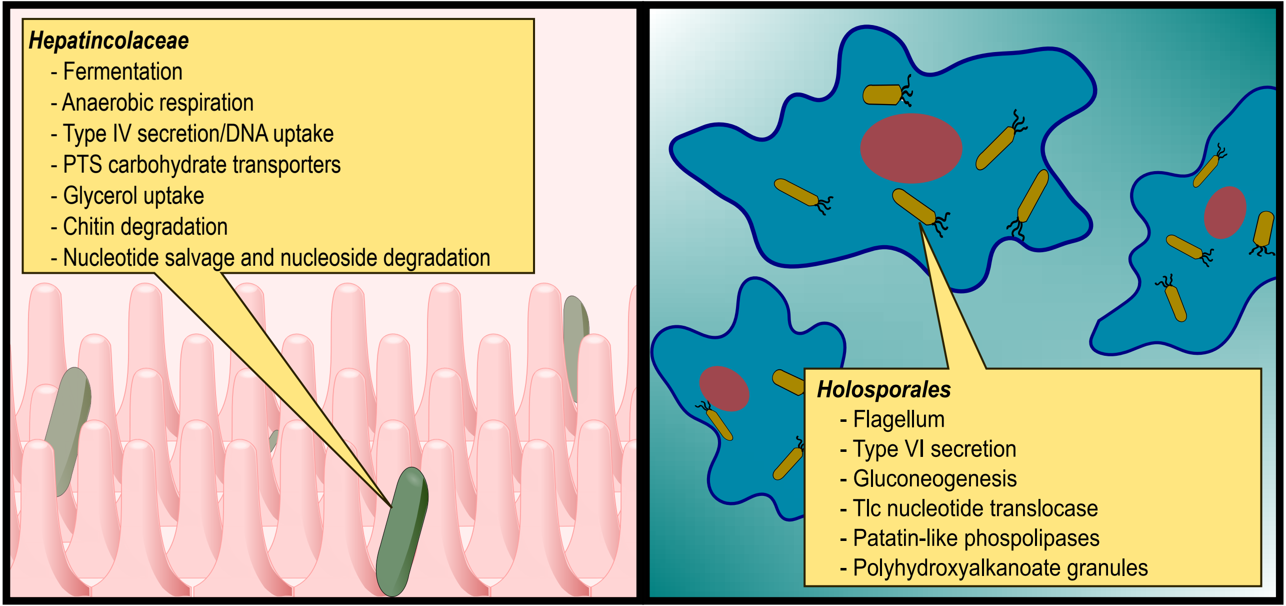

Hepatincolaceae
- Fermentation
- Anaerobic respiration
- Type IV secretion/DNA uptake
- PTS carbohydrate transporters
- Glycerol uptake
- Chitin degradation
- Nucleotide salvage and nucleoside degradation
Holosporales
- Flagellum
- Type VI secretion
- Gluconeogenesis
- Tlc nucleotide translocase
- Patatin-like phospholipases
- Polyhydroxyalkanoate granules
