# Supplementary material for: Hepatincolaceae (Alphaproteobacteria) are Distinct From Holosporales and Independently Evolved to Associate With Ecdysozoa
Source: Environ Microbiol. 2025 Jan 10;27(1):e70028. doi: 10.1111/1462-2920.70028 (PMC11724238; doi:10.1111/1462-2920.70028)
Supplement: Supplementary file 21 — Text S1. Detailed description of the metabolic features of the ‘Candidatus Hepatincolaceae’ in comparison with host‐associated and free‐living relatives. [file EMI-27-e70028-s019.pdf]

Supplementary text 1: detailed genome comparisons

Carbohydrate and energy metabolism

The *Hepatincolaceae* are able to perform most of the core reactions in carbohydrate and energy metabolism, though with some limitations and peculiarities, in comparison with the other host-associated bacteria herein analysed.

All the *Hepatincolaceae* are able to perform glycolysis, in most cases already starting from exose sugars (glucose-6-phosphate) with the exception of the symbiont of *Strigamia*, which may only process directly trioses (glyceraldehyde-3-phosphate). Some among these and other missing genes (see below) in the latter symbiont as compared to other *Hepatincolaceae* may be explained by the probably incomplete status of its genome assembly (Supplementary Table 6, 7). Such repertoire is equivalent to the symbiont of *Haliotis* (which though lacks enolase) and to the richest ones among *Holosporales,* namely *Bealeia* among *Holosporaceae*, as well as the *Caedimonadaceae* and the *Paracaedibacteraceae.* Partial exceptions are *Nucleicultrix* lacking the ability to isomerise the trioses (glyceraldehyde-3-phosphate and glycerone phosphate), and *Paracaedibacter* lacking the initial isomerising step between the exoses (glucose-6-phosphate and fructose-6-phosphate). For what concerns the other investigated *Holosporaceae*, *Hepatobacter* can only perform the isomerisations between the exoses and between the trioses, while *Holospora* is fully devoid of this pathway.

The *Hepatincolaceae* are equipped only with some of the steps that are specific of gluconeogenesis with respect to glycolysis, namely the initial ones that are also part of Krebs cycle (see below). Specifically, *Hepatincola* spp. can convert malate to phosphoenolpyruvate, the symbiont of *Labiotermes* can convert it to oxaloacetate, the symbiont of *Strigamia* can do both conversions, while *Tardigradibacter* neither. The symbiont of *Haliotis* has a poor competence in those steps, but different from the *Hepatincolaceae*, being able to perform only the conversion of pyruvate to phosphoenolpyruvate among the steps that are typical of them, but it can also dephosphorylate fructose-1,6-phosphate. On the other hand, among the *Holosporales* the richest one is *Bealeia*,

being equipped with all gluconeogenesis-specific steps, thus being able to perform the whole pathway. *Holospora* and *Hepatobacter* can convert malate into phosphoenolpyruvate, all the *Caedimonaceae* and *Paracaedibacteraceae* can convert pyruvate into oxaloacetate and pyruvate, but only *Odyssella* can get phosphoenolpyruvate.

The *Hepatincolaceae* have most genes for the non-oxidative phase of the pentose-phosphate pathway, except for the transaldolase (present in the symbiont of *Haliotis* and in most *Holosporales*). However, the *Hepatincolaceae* may nevertheless "fuel" the pentose phosphate pathway, thanks to the glycolytic enzymes phosphofructokinase and fructose bisphosphate aldolase, which allow performing the sedoheptulose bisphosphate bypass, thus interconverting sedoheptulose bisphosphate into dihydroxyacetone-phosphate and erythrose-4-phosphate (instead of interconverting sedoheptulose bisphosphate and glyceraldehyde-3-phosphate into fructose-6-phosphate and erythrose-4-phosphate, as in the conventional pathway).

Just like the *Holosporales* and the symbiont of *Haliotis*, all the *Hepatincolaceae* (besides the symbiont of *Strigamia*) present the pyruvate dehydrogenase complex, thus allowing the production of acetyl-CoA for fuelling Krebs cycle (when present) or at least biosynthetic pathways, e.g. for lipids.

On the other hand, the Krebs cycle is never complete among *Hepatincolaceae*, which always miss at least the 2-oxoglutarate dehydrogenase. The richest are *Hepatincola* spp. and the symbiont of *Strigamia*. *Hepatincola* spp. present the part of the cycle from succinate to 2-oxoglutarate. The symbiont of *Strigamia* can start a step earlier (from succinyl-CoA), but lacks citrate synthase (and has a different kind of malate dehydrogenase, see above regarding gluconeogenesis). On the other hand, the symbiont of *Labiotermes* can only convert fumarate to oxaloacetate (with NAD+ reduction), while *Tardigradibacter* is fully devoid of the Krebs cycle. Such reductions (or complete losses) of the Krebs cycle are consistent with other bacterial symbionts of eukaryotes, including the symbiont of *Haliotis* (having only the aconitase and the NADH-producing 2-oxoglutarate

dehydrogenase), and some *Holosporales* like *Holospora* (Garushyants *et al.*, 2018), although many other members of this order display a substantially complete Krebs cycle (Giovannini *et al.*, 2024).

All the *Hepatincolaceae* have at least a minimal set for oxidative phosphorylation, namely NADH-ubiquinone oxidoreductase (exploiting at least the NADH obtained during glycolysis), and at least one ubiquinol oxidase employing oxygen as terminal acceptor, specifically the cytochrome o type in *Tardigradibacter*, the cytochrome bd type in the symbionts of *Strigamia* and *Labiotermes*, and both types in *Hepatincola* spp. This indicates probable independent steps of pathway reduction among the *Hepatincolaceae* as compared to their free-living relatives, which would also be convergent as compared to the symbiont of *Haliotis* (having the same set as the symbionts of *Strigamia* and *Labiotermes*) and to the *Holosporales* (typically having also the cytochrome c reductase and oxidase, but never the cytochrome o terminal oxidase).

Besides aerobic respiration, most *Hepatincolaceae* bear the capability to grow also in anaerobic conditions. The symbiont of *Labiotermes* can use nitrate as an alternative terminal electron acceptor thanks to the nitrate reductase, and is also equipped with a narK nitrate/nitrate transporter to interchange nitrate and its reduction product nitrite. This capability is shared with the symbiont of *Haliotis* and several free-living relatives, but with none of the *Holosporales*. An alternative/additional strategy for anaerobic growth in all the *Hepatincolaceae* except the symbiont of *Strigamia* is fermentation. Specifically, *Hepanticola* spp. and *Tardigradibacter* can recycle NADH by reducing pyruvate into lactate. Interestingly, *Hepatincola* spp. (but not *Tardigradibacter*), as well as the symbiont of *Labiotermes* have a lactate permease, possibly involved in the excretion of this by-product. Whereas, thanks to a pyruvate-formate lyase, the symbiont of *Labiotermes* can anaerobically convert pyruvate into formate (excreted via a dedicate transporter) and acetyl-CoA, which can then be converted to acetaldehyde and finally ethanol via the aldehyde-alcohol dehydrogenase. Although these reactions do not allow a net NADH recycling, this is complemented by the capability of this bacterium of anaerobic respiration. While none among *Holosporales* is able to ferment, the symbiont of *Haliotis* is competent for both kinds of

fermentations mentioned above (including formate excretion), while the free-living are only capable of lactic fermentation. Interestingly, the two pyruvate-formate lyases of the symbionts of *Labiotermes* and *Haliotis* have high identities (>70% identity) with different distantly-related bacteria (*Bacillota* and *Betaproteobacteria*, respectively), and are thus probably the result of two different horizontal gene transfer (HGT) events. Moreover, all the *Hepatincolaceae* (as well as the symbiont of *Haliotis* and some among the free-living relatives and the *Holosporales*) can interconvert acetyl-CoA and acetate via phosphate acetyltransferase and acetate kinase. This can either allow using acetate to fuel Krebs cycle in aerobic conditions, or, reversely, producing acetate to be excreted in anaerobic conditions, thereby also obtaining ATP.

The *Hepatincolaceae* are also equipped with a quite rich set of transporters that may enable them to obtain several metabolites from their hosts, including intermediates of the canonical pathways exposed above, as well as several other molecules, which may be further converted in alike intermediates thanks to dedicated enzymes, in order to fuel energetic metabolism (see also paragraphs below regarding capabilities to obtain energy from the degradation of lipids, amino acids, and nucleotides). In particular, the *Hepatincolaceae* have a quite rich set of PTS (phospho-transferase system) sugar transporters. The most widespread are transporters for fructose (present in all the *Hepatincolaceae*), glucose (in all except *Hepatincola* Pdp), and N-acetyl-glucosamine (two different paralogs present, although one is truncated in *Tardigradibacter* and fully absent in the symbiont of *Strigamia*). A PTS transporter for N-acetyl-glucosamine is present also in the symbiont of *Haliotis*, but absent in the free-living relatives. Consistently, all the *Hepatincolaceae* and the symbiont of *Haliotis* are able to process N-acetyl-glucosamine into fructose-6-phosphate (fuelling glycolysis), unlike most free-living relatives and the *Holosporales*. A PTS transporter for mannitol is present in all *Hepatincola* spp. and in the symbiont of *Labiotermes*, as well as in the symbiont of *Haliotis*, and all these bacteria may convert imported mannitol into fructose-6-phosphate via the mannitol-1-phosphate dehydrogenase. The symbionts of *Labiotermes* and *Strigamia* possess

additional PTS system components such as those homologous to those specific for chitobiose/lichenane (in triple copy in the symbiont of *Labiotermes*), for lactose/cellobiose or for ascorbate (the latter only in the symbiont of *Labiotermes*). *Tardigradibacter* has an additional PTS for beta-glucosides. The symbiont of *Haliotis* is overall poorer in PTS, and, together with the those mentioned above, possesses a transporter for mannosyl-glycerate. On the other hand, the free-living relatives and the *Holosporales* are fully devoid of PTS for carbohydrates.

Differently from the symbiont of *Haliotis* and the *Holosporales*, but similarly to many free-living relatives, most *Hepatincolaceae* (all except the symbiont of *Strigamia*) can import glycerol and metabolise it into the glycolytic intermediate dihydroxyacetone-phosphate, via glpD, glpK and glpF. Considering also the presence of pentose-phosphate pathway with the sedoheptulose bisphosphate bypass (which may be alimented by dihydroxyacetone-phosphate), this ultimately means that these *Hepatincolaceae* may use glycerol as primary carbon source.

The symbionts of *Strigamia* and *Labiotermes* can phosphorylate glucose into glucose-6-phosphate by glucokinase, just like the symbiont of *Haliotis* and some free-living relatives, while, differently from the symbiont of *Haliotis* and the *Holosporales*, all the *Hepatincolaceae* except *Hepatincola* spp. can metabolise fructose-1-phosphate towards glycolysis. Differently from the other herein analysed bacteria, except some free-living relatives, all the *Hepatincolaceae* may also isomerise mannose-6-phosphate into fructose-6-phosphate. *Tardigradibacter* has a maltoporin for importing maltose and related compounds. The symbiont of *Labiotermes* can phosphorylate dihydroxyacetone, thus converting it into an intermediate of glycolysis (and of sedoheptulose bypass). The symbiont of *Labiotermes* also possess several enzymes (in particular homologs of uxuA, uxuC, uxaA, uxaC, and eda) that may enable the metabolism into glycolytic intermediates of metabolites such as fructuronate/galacturonate/glucuronate, which may be derived from partially host-digested plant-derived materials such as pectin (Renard *et al.*, 1999). Differently from the symbiont of *Haliotis*, the *Hepatincolaceae* cannot metabolise galactose directly, but only UDP-galactose (a capability shared with many among their free-living relatives and the *Holosporales*).

Moreover, *Tardigradibacter* and the symbiont of *Labiotermes* can metabolise acetoacetate to acetyl-CoA through acetoacetyl-CoA transferase and acetyl-CoA acetyltransferase (the former shared with a few free-living relatives, the second with more of those relatives and also with some *Holosporales*). However, the *Hepatincolaceae* cannot use glycogen or polyhydroxyalkanoate granules as carbon storages, unlike, respectively, the symbiont of *Haliotis* and few free-living relatives, or several among free-living relatives and *Holosporales*.

Finally, all the arthropod-associated *Hepatincolaceae* (i.e., all except *Tardigradibacter*) have some proteins that may be active against chitin or chitin-derivatives, such as chitinase II-like domain proteins (several genes in *Hepatincola* spp. and in the symbiont of *Labiotermes*), a lytic polysaccharide monooxygenase (which may be active on chitin or cellulose) in the symbiont of *Strigamia*, and a chitin deacetylase in the symbionts of *Strigamia* and *Labiotermes*. All these proteins are distantly related to each other (identities below 45% besides putative orthologs of *Hepatincola* spp.) and to the chitinase II domain of "*Ca*. Hepatobacter" among *Holosporales*, thus probably representing results of independent HGT events.

Metabolism of lipids, phospholipids, peptidoglycan, and lipopolysaccharide

As typical also in the other bacteria herein analysed, all the *Hepatincolaceae* are competent in producing the main components of the typical Gram-negative cell membranes and wall. Specifically, they can synthesise lipids (including initiation and elongation) and phospholipids, with the possible exception of the symbiont of *Strigamia*, having only a single subunit (AccA) of the acyl-CoA carboxylase, and the two final steps of elongation (FabZ and FabI). Moreover, similarly to some free-living relative and to the *Holosporales*, all *Hepatincola* spp. (but not other *Hepatincolaceae* or the symbiont of *Haliotis*) have a fadB-like protein, which might be involved in fatty acid oxidation, thus allowing getting energy (in the form of NADH) from such compounds.

Most *Hepatincolaceae* can synthesise N-acetyl-glucosamine (precursor of both peptidoglycan and lipopolysaccharide), specifically only *Hepatincola* spp. and *Tardigradibacter* bear the full pathway

from fructose-6-phosphate, while the first gene is missing in the symbiont of *Labiotermes*, and the symbiont of *Strigamia* has only the last one (*glmU*). All the *Hepatincolaceae* can synthesise peptidoglycan (although the symbiont of *Strigamia* lacks murJ), including crosslinks, but lacking glutamate racemase (just like most *Holosporales*), and can produce lipolysaccharide (although the symbiont of *Strigamia* lacks kdsA and lpxB).

In addition, most *Hepatincolaceae* have the complete methylerythrytol phosphate pathway for the synthesis of isoprenoids, the only exception being the symbiont of *Strigamia*, only having *ispE* and *ispG* genes. All the other *Hepatincolaceae*, just like other *Alphaproteobacteria* (Testa *et al.*, 2006) including their free-living relatives (but unlike *Holosporales* and the symbiont of *Haliotis*) present ispD and ispF of this pathway fused in a single polypeptide. In *Tardigradibacter* this gene contains a rather large insertion (~190 aa) in its IspD domain, which may reduce/impair its functionality. Interestingly, this bacterium bears an additional *ispD* gene, with best hits, though at low identity (<50%) on non-alphaproteobacterial sequences. This suggests that the latter gene could have been gained by HGT, and has possible replaced at least partially the function of the former gene. No isopentenyl-diphosphate isomerase is present in the *Hepatincolaceae,* in the symbiont of *Haliotis*, or in their free-living relatives, while being quite rare also in the *Holosporales.*

Metabolism of nucleotides

Among the herein investigated host-associated bacteria, the nucleotide synthesis abilities of the *Hepatincolaceae* are somehow intermediate between the symbiont of *Haliotis* and the *Holosporales*. Regarding purines, while the symbiont of *Haliotis* and the free-living relatives can get these molecules from the pentose-phosphate pathway intermediate ribose-5-phosphate, the *Hepatincolaceae* can at most perform the final biosynthetic steps. Specifically, the symbiont of *Labiotermes* may produce GMP from inosinate, while all the *Hepatincolaceae* except *Tardigradibacter* can use the same compound to obtain AMP. On the other hand, the *Holosporales* are typically unable to perform these reactions and should derive such nucleotides directly from

their hosts. All *Hepatincolaceae* have adenylate and guanylate kinases (the latter besides the symbiont of *Strigamia*) for "recharging" with phosphates.

The capability of synthesising pyrimidines among the *Hepatincolaceae* starts as well from some intermediate compounds. Specifically, it starts from orotidine-5-phosphate in *Hepatincola* spp. (and possibly the symbiont of *Strigamia*, though it lacks the enzymes for several the following reactions, including synthesis of CTP from UTP), and from the following compound (i.e., UMP) in all the others. The symbiont of *Haliotis* has an equivalent set to *Hepatincola* spp. for those reactions, while the *Holosporales* may only convert UTP into CTP. All the *Hepatincolaceae* can synthesise thymine from dUTP (although the symbiont of *Strigamia* lacks the dUTP diphosphatase) or from dCMP (i.e., obtaining dUMP via deamination).

All the *Hepatincolaceae* can convert ribonucleotides into deoxyribonucleotides via a thioredoxin/glutaredoxin-dependent (absent in *Tardigradibacter*) and/or cobalamin-dependent (absent in the symbiont of *Strigamia*) reductase. The first type of reductase is present in the symbiont of *Haliotis* and in most *Holosporales*, while few *Holosporales* have also (or exclusively, in case of "*Ca*. Bealeia" and "*Ca*. Finniella") the second type. Moreover, the symbiont of *Labiotermes* has an additional anaerobic ribonucleoside-triphosphate reductase, just like the symbiont of *Haliotis* and some free-living relatives.

The *Hepatincolaceae* have several proteins involved in importing nucleosides/nucleotides, such as a nupC nucleoside permease (present in all the *Hepatincolaceae*, and widespread in the *Holosporales,* but absent in the symbiont of *Haliotis*), tsx outer membrane and NCS2 nucleoside permeases (both the latter two types are present in all the *Hepatincolaceae* besides the symbiont of *Strigamia*, but in *Tardigradibacter* NCS2 seems truncated), a cytosine permease in the symbiont of *Strigamia*, and a xanthine/uracil permease in all *Hepatincola* spp. and in the symbiont of *Labiotermes*. Whereas, the tlc translocases for nucleotides, typical in *Holosporales* and other host-associated bacteria, are fully absent in the *Hepatincolaceae* and the symbiont of *Haliotis* (as well as in their free-living relatives).

Consistently with the potential to import several nucleotide by-products from their host digestive tract, the *Hepatincolaceae* present some capabilities of nucleotide salvage. These include adenine and guanine/hypoxanthine phosphoribosyltransferases (both present in the symbiont of *Haliotis* and among the free-living relatives, while only the latter is present among the *Holosporales*), as well as, only in the symbiont of *Strigamia*, a hypoxanthine phosphoribosyltransferase, shared with few free-living relatives. Moreover, all the *Hepatincolaceae* are able to degrade (deoxy)nucleosides via the deoABCD pathway, allowing to fuel glycolysis by their glucidic part (although the symbiont of *Strigamia* lacks deoA). On the other hand, *Tardigradibacter* has two *deoD* genes with quite high amino acidic identity (>80%), probably representing recent duplicates with possibly different substrate specificity. The symbiont of *Haliotis* has the full pathway as well, while the free-living relatives lack the *deoD* gene, and *Holosporales* are fully devoid of this degradation pathway. In addition, all *Hepatincolaceae* and the symbiont of *Haliotis* can deaminate cytidine into uracyl, which may forage either the salvage or the degradation pathway.

Metabolism of amino acids

Biosynthetic abilities for amino acids among the *Hepatincolaceae* are even scarcer than for nucleotides. They can all produce alanine from cysteine, and glycine from serine. *Hepatincola* spp. and the symbiont of *Labiotermes* can interconvert aspartate into glutamate, and fumarate into aspartate (with the reverse reaction that might provide an intermediate of the Krebs cycle for energy production in the reverse sense). The symbionts of *Strigamia* and *Labiotermes* can produce glutamate from 2-oxoglutarate. Only *Tardigradibacter* can obtain lysine from aspartate, while the other *Hepatincolaceae* have only partial sets for the enzymes of the final steps. In particular, all can obtain diaminopimelate (required for peptidoglycan synthesis) from L,L-diaminopimelate. Moreover, all the *Hepatincolaceae* (including *Tardigradibacter* that also has a lysA protein) have a LysA family protein, which may complement the lack of lysA in the last step of lysine biosynthesis, namely conversion of diaminopimelate into lysine. The symbiont of *Haliotis* and the free-living

relatives have the whole pathway for lysine biosynthesis, while most *Holosporales* can only produce diaminopimelate, but, differently from the *Hepatincolaceae*, all using a argD-like rather than a serC-like protein for the N-succinyldiaminopimelate aminotransferase step.

Hepatincola spp. and the symbiont of *Labiotermes* can produce homocysteine from cystathionine, but are unable to process it into methionine. All the *Hepatincolaceae* can synthesise chorismate (although aroE is missing in the symbiont of *Strigamia*), but cannot convert it into the proteinogenic aromatic amino acids. In addition, while no *Hepatincolaceae* can synthesise branched-chain amino acids, the symbiont of *Strigamia* has the terminal broad-specificity ilvE aminotransferase. Conversely, this bacterium lacks the glutamyl-tRNA(Gln) amidotransferase for the synthesis of glutaminyl-tRNA(Gln), which is present in all the other *Hepatincolaceae*. This paucity of amino acid biosynthetic pathways among the *Hepatincolaceae* is consistent with the average *Holosporales*, conversely, the symbiont of *Haliotis* can produce almost all amino acids, with the exceptions of proline and tyrosine.

Consistently with their limited biosynthetic capabilities, the *Hepatincolaceae* have a quite rich set of transporters which may be involved in the import of amino acids, as well as degradative enzymes that may enable the usage of these compounds not only for protein biosynthesis, but also for energy production. These include broad-specificity systems, such as the oppBCD system for the transport of oligopeptides in *Tardigradibacter* and the symbiont of *Labiotermes* (present also in the symbiont of *Haliotis*), or several dicarboxylate/amino acid:cation family symporters, which are widespread in multiple copies among the *Hepatincolaceae* as well as among the *Holosporales*, but absent in the symbiont of *Haliotis* and present only in a single copy in *Thalassospira* among the free-living relatives. Additional systems with higher specificity could be identified, such as tcyP for cystine uptake and a methionine transporter in all the *Hepatincolaceae*. All the *Hepatincolaceae* except *Tardigradibacter* also have a putP-like proline importer, just like the symbiont of *Haliotis* and several among their free-living relatives and the *Holosporales.* On the other hand, all *Hepatincola* spp. and the symbiont of *Strigamia* may get energy from proline degradation, thanks to a putA-like

dehydrogenase (which reduces quinones), present as well in the free-living relatives and the *Holosporales*.

All *Hepatincola* spp. have an arginine translocator and, similarly to the symbiont of *Labiotermes*, may convert this compound into citrulline via the arginine deiminase, and this product may be then converted by the ornithine carbamoyltransferase into carboamyl phosphate and ornithine. The first may be processed by the carbamate kinase to synthesise ATP directly (in *Hepatincola* spp. and the symbiont of *Labiotermes*), while the second may be also metabolised to get energy, being converted (only in *Hepatincola* spp.) via ornithine cyclodeaminase into proline, which may then be further oxidised to reduce ubiquinone (see above).

Hepatincola spp. (as well as several among the free-living relatives and the *Holosporales*) may get energy for alanine oxidation, while all *Hepatincola* spp. and the symbiont of *Labiotermes*, as well as the symbiont of *Haliotis* and some free-living relatives, may get it from serine degradation. Finally, the symbionts of *Strigamia* and *Labiotermes* have a Glu/Leu/Phe/Val dehydrogenase homolog (present also in some free-living relatives), which may allow getting energy from the oxidation of several of such amino acids.

Metabolism of cofactors

Differently from their very limited abilities to produce nucleotides and amino acids, the *Hepatincolaceae* have the capability to synthesise *de novo* or salvage several cofactors. The symbiont of *Strigamia* is the only one among *Hepatincolaceae* that can synthesise biotin, whereas *Tardigradibacter* has a bioY transporter. The symbiont of *Haliotis* can synthesise biotin as well, while among *Holosporales* there is an inverse correlation between the presence of biosynthesis or of bioY (Giovannini *et al.*, 2024). All the *Hepatincolaceae* can synthesise CoA from pantothenate (although the symbiont of *Strigamia* lacks the enzymes for the first and last steps coaA and coaE, while *Hepatincola* spp. and *Tardigradibacter* lack coaBC). This repertoire is a subset of the one of the symbiont of *Haliotis* and some among *Holosporales*, which can also produce pantothenate from

2-keto-isovalerate (an intermediate of the synthesis of the amino acid valine). All the *Hepatincolaceae* can synthesise lipoate (although the symbiont of *Strigamia* lacks the enzyme for the last step), similarly to the free-living relatives, the symbiont of *Haliotis* and most *Holosporales*. Just like the other bacteria herein analysed, all the *Hepatincolaceae* can also produce iron-sulfur clusters and glutathione.

All the *Hepatincolaceae* are competent for the NAD salvage pathway (although nadD and pcnB are missing in the symbiont of *Strigamia*), but not for its *de novo* synthesis. On the other hand, NAD *de novo* synthesis/salvage pathways are rare and anyway incomplete in the *Holosporales* and the symbiont of *Haliotis* (only the last *nadE* gene is present in the *Holosporales* bacterium "*Ca*. Finniella", while the last two, *nadD* and *nadE,* in the symbiont of *Haliotis*, both genes being shared between synthesis and salvage pathways). Consistently, differently from other organisms examined, all the *Hepatincolaceae* and the symbiont of *Haliotis* have a pnuC transporter, which may enable the uptake of NAD by-products as basis for its salvage. Otherwise, just like the symbiont of *Haliotis* and as typical among *Holosporales*, all the *Hepatincolaceae* can phosphorylate NAD into NADP. Among the *Hepatincolaceae*, only *Tardigradibacter* has a NAD/NADP transhydrogenase, just like several free-living relatives and *Holosporales.* While all the *Hepatincolaceae*, as well as the symbiont of *Haliotis*, can convert riboflavin into FAD, only the symbiont of *Strigamia* can produce riboflavin from GTP. On the other hand, *Holosporales* are variable in FAD synthesis abilities, and only some are competent. Regarding folate, all the *Hepatincolaceae* can obtain it from dihydropteroate, but, differently from the symbiont of *Haliotis* and few *Holosporales*, cannot synthesise the latter compound from GTP and chorismate.

All the *Hepatincolaceae* can synthesise pyridoxal phosphate with the *pdxAJH* genes, just like some *Holosporales*, while the symbiont of *Haliotis* may obtain the same compound with the *pdxST* genes. Moreover, just like the symbiont of *Haliotis* and most *Holosporales*, all the *Hepatincolaceae* can synthesise ubiquinone, and this pathway is a potential downstream processing of chorismate, in the absence of aromatic amino acid synthesis. The symbiont of *Labiotermes* has the capability for

thiamine diphosphate salvage, while other *Hepatincolaceae* can only perform the last step of conversion of thiamine phosphate into thiamine diphosphate. The symbiont of *Haliotis* cannot produce *de novo* this cofactor but can salvage it, while only a few among *Holosporales* can produce/salvage it. Among the *Hepatincolaceae*, only the symbiont of *Labiotermes* can synthesise molybdopterin and molybdenum cofactor, while the symbiont of *Haliotis* and many free-living also do so, but the *Holosporales* do not. This pattern is consistent with the presence/absence of the molybdoenzyme nitrate reductase in the same organisms (see above). On the other hand, consistently with the lack of cytochrome c, none among *Hepatincolaceae* can synthesise heme, differently from several *Holosporales* and the symbiont of *Haliotis* (although the latter lacks cytochrome c oxidase and reductase, too, see above).

Secretion, motility, and other interactions with the host

All the *Hepatincolaceae* possess the core Sec translocon and the components of co-translational transport (ftsY, ffh), although few genes are missing in some members, in particular those with the lowest BUSCO scores (s*ecD* missing in *Hepatincola* Pdp and the symbiont of *Strigamia*, *secE* in *Hepatincola* Av. *secG* in the symbiont of *Strigamia*, *yaiC*, *secA* and *secF* in *Hepatincola* Pdp). The *Hepatincolaceae* lack the post-translational transport component (*secB*), which is present in the symbiont of *Haliotis* and most *Holosporales* (as well as in the free-living relatives). On the other hand, all the *Hepatincolaceae* possess the Sec-independent post-translational Tat secretion system, just like the symbiont of *Haliotis*, while only a minority of *Holosporales* have it. Similarly to the typical representatives of the other groups of bacteria analysed, the *Hepatincolaceae* also possess lol and bam systems, for lipoprotein and outer membrane protein transport, respectively.

Differently from many *Holosporales*, neither *Hepatincolaceae* nor the symbiont of *Haliotis* have type II secretion/type IV pilus. Moreover, the *Hepatincolaceae* have no flagellum/type III secretion system or chemotaxis, which are present in the symbiont of *Haliotis* and common among *Holosporales*. While the vast majority of *Holosporales* has a type VI secretion system (George *et*

al., 2020), this is absent in the *Hepatincolaceae,* the symbiont of *Haliotis* and their close free-living relatives.

However, differently from all the other organisms herein analysed, some members of the *Hepatincolaceae*, namely *Hepatincola* Av, *Tardigradibacter* and the symbiont of *Strigamia*, possess genes homologous to components of the type IV secretion system. Specifically, Hepatincola Av has in single copy *virB9*, *virB10*, *virb8*, *virD4*, *virB11*, *virB4*, and *virD2*. The symbiont of *Strigamia* lacks *virB4* but shares all others, each in double copy. On the other hand, *Tardigradibacter* is equipped with a smaller repertoire, having only *virB11*, as well as *virB4* and *virD2*, but both divided into multiple consecutive ORFs and thus potentially pseudogenised. The presence of *virD2* among the *Hepatincolaceae* is noteworthy, as this DNA relaxase is implied in DNA transfer (Christie, 2016).

We could also identify some proteins bearing repeat classes that have been previously indicated as potentially involved in bacterial-host interactions, with several lineage-specific variations. Among the *Hepatincolaceae*, we detected proteins with ankyrin repeats (multiple proteins per each genome) and with tetratricopeptide repeats (only in the symbiont of *Labiotermes*). Ankyrin-repeat proteins are also present among the *Holosporales,* abundant in the symbiont of *Haliotis*, while rare in the free-living relatives of the *Hepatincolaceae*. Tetratricopeptide-repeat proteins are diffused in all other groups of investigated organisms. Only some among the *Holosporales* bear proteins with leucine-rich repeats, while pentapeptide repeats could be found in some free-living bacteria and in "*Ca*. Bealeia" among the *Holosporales*.

Other genomic features

All the *Hepatincolaceae* have mismatch repair (although mutL is missing in the symbiont of *Strigamia*), nucleotide excision repair, single-strand break homologous recombination (although recJ is missing in the symbiont of *Strigamia*), and addAB double-strand break homologous recombination. This repertoire is consistent with the symbiont of *Haliotis* and most *Holosporales*.

In addition, the *Hepatincolaceae* bear several of DNA glycosylases involved in base-excision-repair, such as a DNA-formamidopyrimidine glycosylase, a uracil-DNA glycosylase, and multiple 3-methyladenine DNA glycosylases, present in all members, or alkA, present in *Hepatincola* spp. and the symbiont of *Labiotermes*, and alkD, present only in the latter. *Tardigradibacter* and the symbiont of *Strigamia* also have a rarA protein, just like some members of the *Holosporales* and the symbiont of *Haliotis*. This protein might be involved in response to stalled replication forks.

Differently from most *Holosporales*, and just like the symbiont of *Haliotis* and the free-living relatives, the *Hepatincolaceae* do not have any patatin-like phospholipases, which have been implied in the vacuolar escape in intracellular bacteria.

Among the *Hepatincolaceae*, *Tardigradibacter* has *betA* and *betB* genes, involved in the synthesis of the osmoprotectant glycine betaine from choline (Nau-Wagner *et al.*, 2012). Interestingly, the symbiont of *Strigamia* has only a truncated (C-terminal, with ~30% length of the counterpart in *Tardigradibacter*) *betA* gene, while no other member of the *Hepatincolaceae*, nor the symbiont of *Haliotis* or any among *Holosporales* has either gene. Both genes are present in most of the free-living relatives, *betB* also in multiple copies. Intriguingly, in *Tardigradibacter* those genes can be tentatively linked to the peculiar anhydrobiotic cycle of its tardigrade host, as compared to those of the other *Hepatincolaceae*.

Hepatincola Pp and Av, as well as the symbionts of *Labiotermes* and *Strigamia*, have several components of CRISPR/Cas systems.

Many members of *Hepatincolaceae* possess several phage genes, possibly organised in prophages. In *Tardigradibacter*, two putative prophages are present, showing high reciprocal sequence identity (indicative of homology), and corresponding to two genomic regions containing a high number of repeats (each repeat would correspond to an identical subregion in the two prophages; Supplementary Figure 3). These genes encode for multiple phage components, including head, tail, baseplate, integrase, terminase, and phage D protein, supporting them being complete prophages. Many homologs of the prophage genes of *Tardigradibacter* could be found among *Hepanticola*

spp., especially in *Hepatincola* Av and Pdp, where homologs are concentrated in two genomic portions, corresponding to those labelled as "prophage regions" 1 and 3 by (Dittmer *et al.*, 2023). Consistently, hits corresponding to the respective "prophage region 3" were also found in *Hepatincola* Pp. Extensive homologies were also found with genes of the symbiont of *Strigamia*, many of which were neighbouring to each other, but in this case the fragmented nature of the genome assembly prevented a clear identification of potential prophages. Despite being highly divergent in sequence, those phage genes of the *Hepatincolaceae* have relatively higher identities with their counterparts in the members of the same family, rather than with those of other bacterial lineages. Taken together, these data are compatible with possibility that such prophages were ancestral in the *Hepatincolaceae*.

Curiously, one of the two prophages in *Tardigradibacter* encodes for two type II toxin-antitoxin systems. For only one of the antitoxins we could detect a homolog among the *Hepatincolaceae*, namely in *Labiotermes*.

References

Christie, P.J. (2016) The Mosaic Type IV Secretion Systems. *EcoSal Plus* **7**:.

Dittmer, J., Bredon, M., Moumen, B., Raimond, M., Grève, P., and Bouchon, D. (2023) The terrestrial isopod symbiont “Candidatus Hepatincola porcellionum” is a potential nutrient scavenger related to Holosporales symbionts of protists. *ISME Commun* **3**: 18.

Garushyants, S.K., Beliavskaia, A.Y., Malko, D.B., Logacheva, M.D., Rautian, M.S., and Gelfand, M.S. (2018) Comparative Genomic Analysis of Holospora spp., Intranuclear Symbionts of Paramecia. *Front Microbiol* **9**: 738.

George, E.E., Husnik, F., Tashyreva, D., Prokopchuk, G., Horák, A., Kwong, W.K., et al. (2020) Highly Reduced Genomes of Protist Endosymbionts Show Evolutionary Convergence. *Curr Biol* **30**: 925-933.e3.

Giovannini, M., Petroni, G., and Castelli, M. (2024) Novel evolutionary insights on the interactions of the Holosporales (Alphaproteobacteria) with eukaryotic hosts from comparative genomics. *Environ Microbiol* **26**: e16562.

Nau-Wagner, G., Opper, D., Rolbetzki, A., Boch, J., Kempf, B., Hoffmann, T., and Bremer, E. (2012) Genetic Control of Osmoadaptive Glycine Betaine Synthesis in Bacillus subtilis through the Choline-Sensing and Glycine Betaine-Responsive GbsR Repressor. *J Bacteriol* **194**: 2703–2714.

Renard, C.M., Crépeau, M.J., and Thibault, J.F. (1999) Glucuronic acid directly linked to galacturonic acid in the rhamnogalacturonan backbone of beet pectins. *Eur J Biochem* **266**: 566–574.

Testa, C.A., Lherbet, C., Pojer, F., Noel, J.P., and Poulter, C.D. (2006) Cloning and expression of IspDF from Mesorhizobium loti. Characterization of a bifunctional protein that catalyzes non-consecutive steps in the methylerythritol phosphate pathway. *Biochimica et Biophysica Acta (BBA) - Proteins and Proteomics* **1764**: 85–96.
